# Validating the standard model of diffusion MRI in white matter with numerical substrates

**DOI:** 10.1162/IMAG.a.1385

**Published:** 2026-10-15

**Authors:** Jasmine Nguyen-Duc, Quentin Uhl, Rita Oliveira, Jonathan Rafael-Patiño, Ileana O. Jelescu

**Affiliations:** Department of Radiology, Lausanne University Hospital (CHUV), Lausanne, Switzerland; Faculty of Biology and Medicine, University of Lausanne (UNIL), Lausanne, Switzerland; Signal Processing Laboratory 5 (LTS5), Ecole Polytechnique Fédérale de Lausanne (EPFL), Lausanne, Switzerland

**Keywords:** diffusion MRI, biophysical models, white matter

## Abstract

The non-invasive estimation of intra- and extracellular microstructural parameters using biophysical models has been a major focus in brain microstructure imaging with MRI. The Standard Model (SM) of diffusion in white matter (WM) provides a unifying framework for various modelling approaches, representing axons as impermeable sticks embedded within a locally anisotropic extra-axonal space. However, the SM relies on simplifying assumptions that may not hold in realistic WM tissue, as they do not take into account axonal undulations, beading, the presence of glial cells, or membrane permeability. In this work, we investigate how SM parameter estimates, derived using the Standard Model Imaging (SMI) framework, behave when the model is applied to realistic numerical WM substrates generated by the CATERPillar tool. Specifically, we vary (i) axonal morphological features such as beading and undulations, (ii) axonal packing density, (iii) orientation dispersion, (iv) membrane permeability of axons and astrocytes separately, (v) myelin volume fraction, and (vi) diffusion time. In each part of the analysis, different noise levels are introduced. Our findings enable users to identify potential biases introduced by varying conditions and to adjust their interpretations accordingly.

## 1 Introduction

Mapping tissue microstructure using diffusion magnetic resonance imaging (dMRI) has caught considerable attention from the scientific community. Its non-invasive application, view of intact *in situ* tissue, wide field of view, and its relatively fast and cheap acquisition make it an excellent choice for studying the brain *in vivo* (Alexander et al., 2019). The underlying rationale is that dMRI signals reflect water diffusion within biological tissues, where diffusion trajectories are affected by cellular structures, such as cell membranes, making the signals sensitive to tissue microstructural organisation.

To investigate microstructural properties using dMRI, various approaches have been developed, with Diffusion Tensor Imaging (DTI) (Basser et al., 1994) being the most widely used. Its extension, diffusion kurtosis imaging (DKI) (Jensen et al., 2005), captures non-Gaussian diffusion and provides additional metrics, yet both remain limited in specificity (Jelescu, Zurek, et al., 2016). To overcome this, there has been an increasing interest in biophysical tissue models (Coelho et al., 2022). The forward problem consists of predicting diffusion signals analytically in simple known tissue geometries (Kiselev, 2017). To solve the inverse problem, the analytical equations of the model, which describe relevant geometric and diffusion-related characteristics, are fit to diffusion signals (Alexander et al., 2019; Jelescu et al., 2020; Novikov et al., 2019; G. J. Stanisz et al., 1997). However, the inverse problem is inherently ill-posed as multiple tissue configurations can yield similar diffusion signals (Coelho et al., 2019; Jelescu, Veraart, et al., 2016; Novikov, Veraart, et al., 2018). An additional complexity arises due to the relatively small size of biological cells (1–10 μm) compared to the typical voxel size (1–2 mm), causing the measured signal to represent a macroscopic average over the entire voxel volume (Kiselev, 2017; Novikov, Kiselev, & Jespersen, 2018). As the diffusion time increases, the random walk of water molecules progressively coarse-grains the underlying microstructure. Fine structural degrees of freedom are averaged out by the diffusing particles and no longer remain encoded in the measurement (Novikov, Kiselev, & Jespersen, 2018; Novikov et al., 2019). Consequently, at clinical diffusion times (> 20 ms), many microstructural features become fundamentally inaccessible to dMRI, which justifies the validity of forward models with simple geometries.

The predominant multi-compartment biophysical model for white matter (WM) is known as the Standard Model (SM) (Novikov, Veraart, et al., 2018; Reisert et al., 2017). In this model, axons (and potentially glial processes) are depicted as impermeable, zero-radius cylinders, commonly termed “sticks”, arranged into locally coherent fibre bundles. Together, these axons define the intra-axonal space (IAS), within which water diffusion is restricted to the axial direction of the individual fibres. On the other hand, diffusion in the extra-axonal space (EAS) of each locally coherent fibre bundle is represented by an axially symmetric diffusion tensor. Within each voxel, the fibres are distributed according to a fibre orientation distribution function (ODF), modelled up to its second- or fourth-order anisotropy invariant (*p*_2_ and *p*_4_). In total, the Standard Model Imaging (SMI) framework estimates at least five parameters: the axonal water fraction *f*, the ODF coefficient *p*_2_, the intra-axonal diffusion coefficient along the principal axonal orientation *D_a_*, the extra-axonal diffusion coefficient perpendicular to the principal axonal orientation *D_e_*_⊥_, and the extra-axonal diffusion coefficient parallel to the principal axonal orientation *D_e_*_∥_. It unifies multiple modeling frameworks (Alexander et al., 2010; Assaf et al., 2004, 2008; Ferizi et al., 2015; Fieremans et al., 2010, 2011; Jespersen et al., 2010, 2018; Kaden et al., 2016; Kroenke et al., 2004; McKinnon et al., 2018; Novikov, Veraart, et al., 2018; Reisert et al., 2017; Scherrer et al., 2016; Sotiropoulos et al., 2012; Wang et al., 2011; Zhang et al., 2012), all based on similar principles but differing in their assumptions (Afzali et al., 2021; Coelho et al., 2022; Jelescu et al., 2020).

The SM is widely accepted within the dMRI community as an appropriate model for WM (Coelho et al., 2022; Jelescu et al., 2022; Liao et al., 2024), largely because myelinated axons are considered the dominant structural components. Despite its usefulness, the SM and biophysical models, in general, still fall short of capturing the true complexity of brain microstructure. While simplifications are necessary to limit the number of parameters to fit, some assumptions require further scrutiny to clarify each model’s validity range. Real axons, for instance, display undulations and calibre variations (H. H. Lee et al., 2020), which can contribute to non-Gaussian diffusion. When such structural irregularities occur at length scales comparable to the diffusion length, the assumption of Gaussian diffusion within compartments may no longer hold. Their influence can be assessed by examining the time dependence of diffusivity (H. H. Lee et al., 2020; Nguyen-Duc et al., 2026). Additional microstructural features (such as myelin, vasculature, membrane permeability (Gardier et al., 2023; Nedjati-Gilani et al., 2017), and the presence of glial cells (Budde et al., 2011; Stolp et al., 2018)) are also often neglected. For the diffusion times typically used in dMRI acquisitions (20–50 ms), the myelin sheath is generally assumed to act as an effective barrier that restricts exchange between the IAS and EAS, thereby minimising its contribution to the diffusion signal. However, unmyelinated axons, which permit water exchange, become increasingly common in pathological conditions such as multiple sclerosis (Jelescu, Zurek, et al., 2016), potentially introducing bias into SM parameter estimates that rely on the assumption of negligible permeability. Greater validation is, therefore, required to establish the range of conditions under which the SM can be reliably applied (Dyrby et al., 2018; Jespersen et al., 2018).

Validating microstructural models by comparing their estimates with histology has been attempted in multiple studies, but this approach is far from straightforward. Indeed, histological tissue processing can unpredictably alter the underlying microstructure (Shepherd et al., 2009), introducing uncertainties that compromise the reliability of the chosen gold standard. An alternative strategy is to validate models by experimentally detecting specific functional forms in animal or human dMRI data (a paradigm borrowed from the physical sciences (Novikov, Kiselev, & Jespersen, 2018)) as was done for the stick model via its characteristic power law ($S\approx \frac{1}{\sqrt{b}}$) (Veraart et al., 2019). Synthetic signals generated directly from the analytical equations of the model of interest can also be compared with experimental data, but this approach cannot capture model failures when the true microstructure diverges from the model’s assumptions. Nonetheless, this technique was adopted for the validation of the Neurite EXchange Imaging (NEXI) model (Uhl et al., 2024) for example. Furthermore, several studies have used physical phantoms (or hardware phantoms) (Chenevert et al., 2011; Fieremans & Lee, 2018; Fieremans et al., 2008; Malyarenko et al., 2016), which incorporate an NMR-visible liquid alone or combined with NMR-invisible materials to produce well-defined microstructural properties. Physical phantoms come with several drawbacks, including complex chemical preparation, limited material availability, the need for specialised equipment, and a lack of standardisation (Jelescu & Budde, 2017). In this work, we pursue yet another strategy, which is model validation using numerical simulations. This approach, increasingly popular in recent years, entails generating a numerical substrate (or virtual tissue) mimicking WM and then running Monte Carlo (MC) simulations to model water diffusion and synthesise dMRI signals. The advantage with this approach is that we may verify whether the estimated parameters are accurate relative to known ground truth (GT) features (Jelescu & Budde, 2017). Despite notable progress in realistic substrate generation with various tools (Aird-Rossiter et al., 2025; R. Callaghan et al., 2020; Ginsburger et al., 2019; Nguyen-Duc et al., 2026; Villarreal-Haro et al., 2023; Winther et al., 2024), this technique has so far been applied mainly to simple substrates in which axons are represented as cylinders and lack realism (Balls & Frank, 2009; Drobnjak et al., 2011; Dyrby et al., 2013; Fieremans & Lee, 2018; Fieremans et al., 2008, 2011; Ginsburger et al., 2018; Hill et al., 2021; Lam et al., 2015; Mercredi & Martin, 2018; Nilsson et al., 2009; Ning et al., 2017). Conversely, some studies (Coronado-Leija, Abdollahzadeh, et al., 2024; H. H. Lee et al., 2020; Salo et al., 2021) have used numerical substrates obtained by segmenting electron-microscopy data (or confocal microscopy (Panagiotaki et al., 2010)), which provide highly realistic microstructural representations. However, this approach offers limited control over the substrate, since it is derived from real tissue, and it is considerably more expensive to acquire than generating numerical substrates “from scratch” with the tools mentioned above.

The aim of our study is, thus, to investigate the robustness and sensitivity of the SMI parameter estimates in scenarios that deviate from the SM assumptions using highly realistic numerical substrates. For this, we use the previously developed CATERPillar tool (Nguyen-Duc et al., 2026) for the generation of realistic numerical WM substrates and the Monte Carlo Diffusion and Collision (MC/DC) simulator (Rafael-Patino et al., 2020) for MC simulations. We evaluate the performance of SMI in estimating parameters across substrates with different levels of complexity. To achieve this, separate analyses were performed on various types of substrates, each analysis representing a specific complexity level with a single varying parameter. These included variations in axonal undulation, beading, volume fraction, permeability, orientation dispersion, myelin volume fraction, and the addition of permeable astrocytes. We hypothesise that incorporating complex axon morphologies, permeability, myelin, and astrocytes will challenge the SM assumptions and affect the SMI parameter estimation (Afzali et al., 2021; Budde et al., 2011; Nilsson et al., 2013; Stolp et al., 2018).

## 2 Methods

This study is based entirely on computational simulations and synthetic data. As it did not involve human participants or animal subjects, ethics committee approval was not required.

### 2.1 Growing substrates with CATERPillar

Numerical substrates representing WM tissue were generated using our newly developed and publicly available CATERPillar framework (Nguyen-Duc et al., 2026). These substrates could be populated with various cell types, including myelinated axons, non-myelinated axons, and glial cells (astrocytes, oligodendrocytes, etc.). To enhance biological realism, the cellular morphologies incorporated complex features such as axonal undulations and beading, parametrised to match histological measurements (Nguyen-Duc et al., 2026). For this study, we simulated substrates with a volume of L = (150 $\mu \text{m}{)}^{3}$, using the default parameters summarised in Table 1.

**Table 1.** Default values for substrates generated by CATERPillar.

Parameters	Control names	Value ranges	Default values
Voxel length [μm]	L	[10, ∞]	150
Gamma distribution parameters for axon radius	α	[0, ∞]	4
β	[0, ∞]	0.25
Volume fraction [%]	f	[0, 100]	50
Beading parameters	A	[0, 1]	0.3
	σ	[0, A]	0.1
Undulation parameter	ε	[0, ∞]	0.2
Orientation dispersion	c_2_	[0.75, 1]	1
Number of populations	N	[1, 3]	1
Membrane permeability	κ	[0, ∞]	0

These parameters lead to the creation of realistic axons as demonstrated in Nguyen-Duc et al. (2026). This Gamma Distribution with α = 4 and β = 0.25 yields a mean axonal radius of 1 μm and a mode of 0.75 μm. Setting A = 0.3 and σ = 0.1 for the beading parameters produces a coefficient of variation (COV) in axonal radius of approximately 0.2 along their length. The undulation parameter *ϵ* results in an undulation coefficient of 1.1. The orientation dispersion, here defined as $c_{2}\;=\langle \text{cos}\phi^{2}\rangle$ (φ being the angle between each axon and the main bundle orientation), can be converted to *p*_2_ via the relationship $p_{2}=1.5c_{2}-0.5$. The number of populations determines the number of modelled fibre bundles, which are assumed to be mutually orthogonal.

The methods for growing numerical WM substrates with CATERPillar are described in detail in Nguyen-Duc et al. (2026). Briefly, axonal growth was initiated by assigning each axon a starting point on one plane and an attractor on the opposite plane with a pre-defined volume fraction *f*. Each axon’s mean radius (*R_mean_*) was sampled from a Gamma Distribution with controllable parameters (by default α = 4 and β=0.12 ). During growth, spheres were sequentially added to form a chain of overlapping elements, extending towards the attractor. Undulations were introduced by setting a non-zero value for *ϵ*, a parameter that modulates the strength of attraction towards the target. Increasing *ϵ* weakens this attraction, leading to more pronounced undulations along the axon’s trajectory, as shown in Figure 1. For instance, with ϵ=0.2 , the typical undulation coefficient (or tortuosity defined as the ratio between the total axon length and the straight-line distance between its endpoints) is approximately 1.1. Beading was implemented in a stochastic manner by sampling the radius of each subsequent sphere from a Gaussian distribution at every growth step. The distribution was centred on the radius of the preceding sphere and constrained to lie within $R_{mean}\;\pm A\times R_{mean}$ . The parameter *A* thus set an upper bound to the amplitude of the beading relative to $R_{\text{mean}}$ . On the other hand, σ controlled the spatial frequency of the beading, as the standard deviation of the Gaussian distributions was equal to σ × *R_mean_*. The σ parameter was fixed in the previous version of CATERPillar (Nguyen-Duc et al., 2026), but is now a tunable user-defined parameter. Setting A = 0.3 and σ = 0.1 produced a coefficient of variation (COV) in axonal radius of approximately 0.2 along their length, consistent with measurements from electron microscopy (H. H. Lee et al., 2019; H.-H. Lee et al., 2024). These values were therefore adopted as the default parameters, inducing beading as illustrated in Figure 2.

**Fig. 1.**
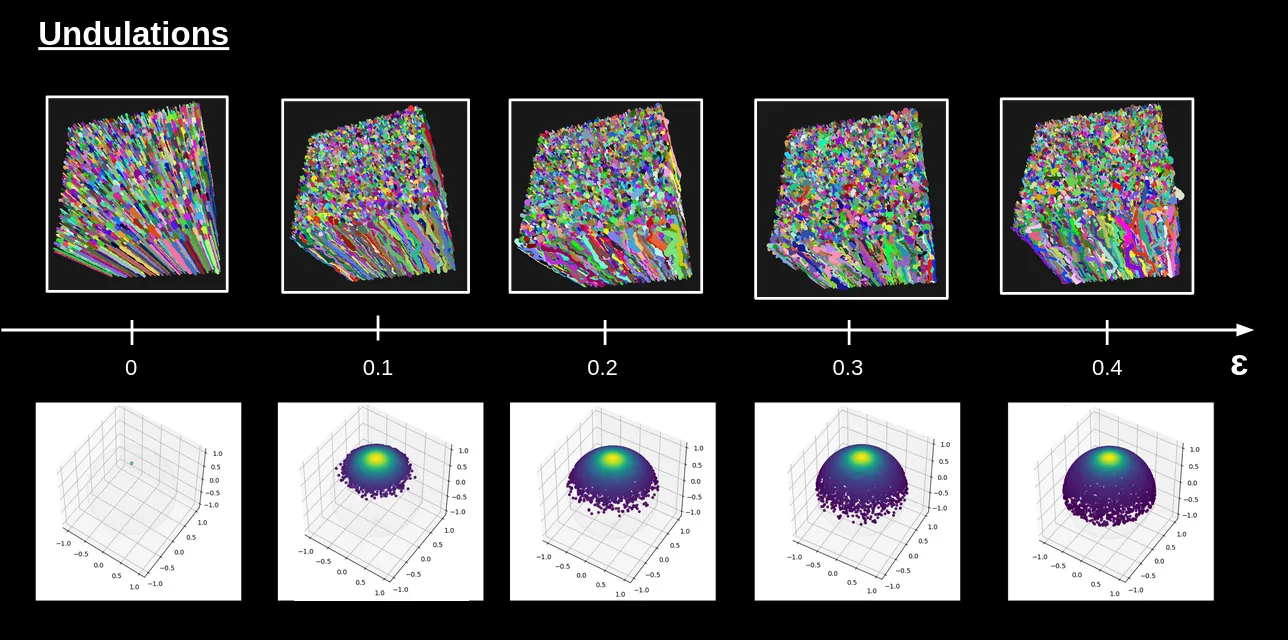
The axonal undulations are governed by the parameter *ϵ*. At each growth step, the angles determining the position of the next sphere are sampled from a Gaussian distribution centred on the axon’s attractor direction. The value of *ϵ* sets the standard deviation of this distribution. When *ϵ* approaches zero, axons extend almost perfectly straight toward their attractor, whereas larger values of *ϵ* introduce increasing deviations, resulting in more pronounced undulations along the axon’s trajectory. The vectors corresponding to the sampled angles are displayed on the spherical surfaces in the last row. A larger value of *ϵ* permits greater deviation of the vectors from the attractor located at the pole.

**Fig. 2.**
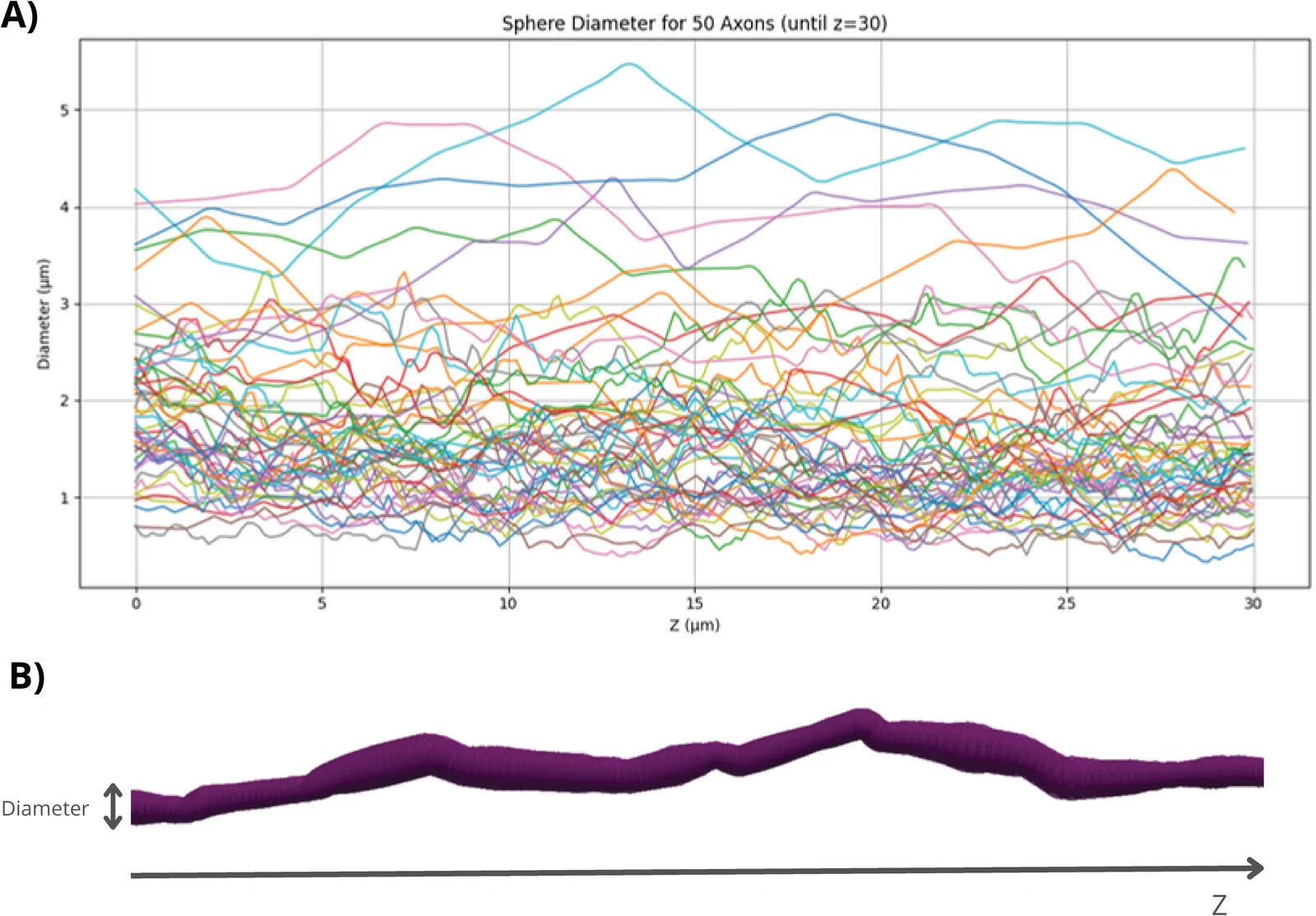
(A) The axonal diameter variation displayed along the main direction (z) for 50 random axons. (B) Illustration of a single axon with varying diameter along its length.

Axonal fanning was also simulated using a Watson distribution parametrised by *c*_2_. In this model, each axon’s attractor was oriented at a specified angle relative to its origin, allowing controlled degrees of fanning. The *c*_2_ parameter could be converted to the SMI orientation dispersion index *p*_2_ through the relationship $p_{2}\;=1.5c_{2}\;-0.5$ . To generate greater overall dispersion, up to three perpendicular fibre populations (*N* = 3) could be created.

After axonal growth was completed, myelin could be introduced by generating inner spheres within the pre-existing ones. The space between the inner and outer spheres represented the myelin sheath and was subsequently excluded from water molecule placement during the MC simulation.

As for glial cells, the somas were placed prior to the axonal growth, while the glial processes (or branches) were grown from the soma towards an assigned attractor after the axons were placed. The mean/standard deviation for the soma radius, number of primary processes, mean/standard deviation for the process length and the volume fractions for the soma and processes can be set by the user, so that the morphological features of the glial cells can match any glial cell of interest. In a part of this work, we populated our substrates with astrocytes, so adapted the morphological parameters so that they matched histological findings, as also previously done in Nguyen-Duc et al. (2026). Examples of astrocytes present in our substrates are shown in Figure 3.

**Fig. 3.**
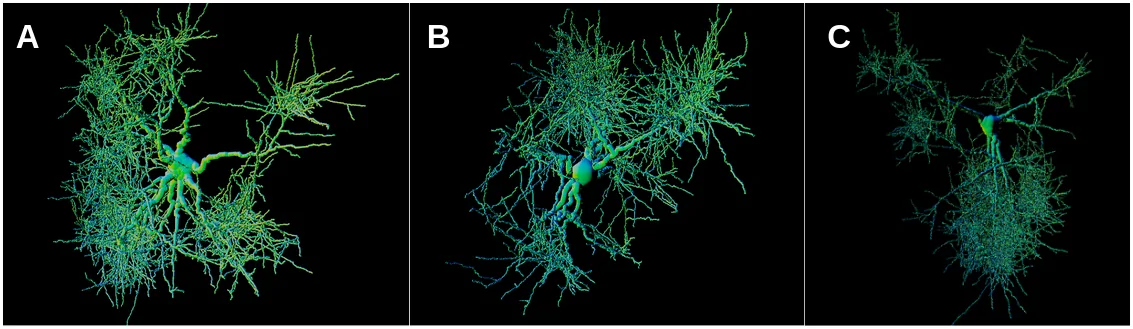
Examples of astrocytes generated with CATERPillar.

### 2.2 Monte Carlo simulations

MC simulations were conducted within the generated voxels with the MC/DC tool (Rafael-Patino et al., 2020). This simulator tool produced diffusion signals from a numerical substrate, neglecting *T*_2_ effects and myelin water, and therefore did not place water particles within the myelin compartment. Simulations used bulk diffusivities of $2.5\mu {\text{m}}^{2}\text{/ms}$ for IAS and $1.5\mu {\text{m}}^{2}\text{/ms}$ for EAS. These diffusivity values were chosen to align with previous studies reporting IAS diffusivities near $2.2\mu {\text{m}}^{2}\text{/ms}$ (Dhital et al., 2019; McKinnon et al., 2018; Veraart et al., 2018) and supporting the condition $D_{a}>D_{e∥}$ (Jespersen et al., 2018; Kunz et al., 2018; McKinnon et al., 2018). Random walkers were initialized in a central ${\left(110\mu \text{m}\right)}^{3}$ region within the ${\left(150\mu \text{m}\right)}^{3}$ cube to avoid edge effects. A step size of 0.1μm (3 times smaller than the smallest sphere diameter) yielded 38, 500 and 23, 100 steps for IAS and EAS, respectively. Simulations used a walker density of 1 walker/μm^3^, which led to a total of 1,330,000 walkers when considering ${\left(110\mu \text{m}\right)}^{3}$. This density of walkers was proven to generate stable signals in Supplementary Figure S1.

Once the trajectories were obtained, we generated synthetic dMRI signals with a pulsed-gradient spin-echo (PGSE) sequence (Stejskal & Tanner, 1965). A wide pulse was utilised to represent clinical scanners, employing Δ=55.5 ms and δ=16.5 ms . The echo time (TE), representing the total duration of the simulation was: TE=Δ+δ+5ms . Sixty unique gradient directions per shell were generated with optimal angular coverage using the method outlined by Caruyer et al. (2013). The b-values utilized were 0, 500, 1000, 2000, 3000, and 5000 s/mm^2^ for SMI estimations. Such high b-values were included as they selectively increase sensitivity to the highly restricted intracellular compartment, and are therefore essential for capturing the microstructural features and morphological irregularities that this work aims to probe. This acquisition design is consistent with Coelho et al. (2022), who showed that optimal SMI acquisition schemes rely on the inclusion of high b-values. Both Gaussian and Rician noise were added to the signal with signal-to-noise ratios (SNRs) of the non-diffusion-weighted signal (b = 0) of 50 and 100, with 1000 random noise realisations per noiseless signal.

### 2.3 Estimating SM parameters using the SMI framework

#### 2.3.1 SM parameters

The objective of this analysis is to evaluate how morphological features that are accounted or unaccounted for explicitly in the model influence the SMI estimations. When fitting SMI to the simulated data, we did not consider the free water (FW) compartment, for which multi-echo time data was needed to solve model degeneracy (Coelho et al., 2022). The fitted microstructural parameters were thus:

1. Axonal Water Fraction (*f*): Represents the relative fraction of MRI signal originating from the IAS. This fraction excludes any myelin water contribution, as myelin water has very short *T*_2_ and is MR-invisible in dMRI due to the typically long echo time used (Jelescu, Zurek, et al., 2016; Mackay et al., 1994; G. Stanisz et al., 1999). When SMI is applied to experimental dMRI data, the relative signal fraction is weighted by the *T*_2_ of each compartment. In our simulations however, we did not place particles inside the myelin layer, and we neglected *T*_2_ relaxation. It is worth noting that the signal fraction *f* is not necessarily equal to the axonal volume fraction (especially in the presence of myelin or compartments with specific *T*_2_ relaxation times).
2. Intra- and Extra-Axonal Diffusivities ($D_{a},D_{e∥},D_{e⊥}$ ): These parameters quantify the diffusion coefficients within both the intra- and extra-axonal spaces. Diffusivity can be measured either parallel (∥) or perpendicular (⊥) to the primary axonal orientation. We assume that the diffusion time corresponding to the SMI estimate for diffusivities is: $\Delta -\frac{\delta }{3}=50\text{ ms}$ .
3. Orientation Distribution Function (fODF): Captures the dispersion of axonal orientations within a voxel. fODF anisotropy is minimally characterized by the *l* = 2 order rotational invariant *p*_2_.

#### 2.3.2 Tool to fit SMI

The TMI Python toolbox (https://nyu-diffusionmri.github.io/DESIGNER-v2/docs/TMI/SMI/) (Ades-Aron et al., 2018; Coelho et al., 2022; Novikov, Veraart, et al., 2018) was used to fit SMI to synthetic dMRI signals. As described in Coelho et al. (2022), the rotational invariants are first computed using a least-squares estimator, then the parameters are obtained using a data-driven machine learning regression. Specifically, a cubic polynomial regression model is employed (Reisert et al., 2017), as it has been demonstrated to be more effective than neural networks for this application (Coelho et al., 2022).

#### 2.3.3 Addition of noise

The synthetic signals were either analysed in their original noise-free state or subjected to Gaussian noise prior to the SMI fitting process. To simulate MRI noise characteristics, a Gaussian noise with a standard deviation of σ was added to the signal as follows:

$$S_{\text{noisy}}\;=S_{noiseless}\;+\epsilon_{\text{re}}$$ (1)

where *S_noiseless_* represents the noise-free signal, and $\epsilon_{\text{re}}\;\sim N\left(0,\sigma^{2}\right)$ is the Gaussian noise component. The noise level σ was determined by the target SNR, defined as $\text{SNR}=S_{0}\text{/}\sigma$ , where $S_{\text{0}}$ is the non-diffusion-weighted signal intensity. We tested two SNR levels of 50 and 100.

Rician noise was also added to the noiseless signal. In this case, independent Gaussian noise was added to the real and imaginary channels of the complex signal independently. The resulting noisy magnitude signal $S_{\text{noisy}}$ was then computed as:

$$S_{\text{noisy}}=\sqrt{{\left(S_{noiseless}\;+\epsilon_{\text{re}}\right)}^{2}\;+\epsilon_{\text{im}}^{2}}$$ (2)

where $\epsilon_{\text{re}},\epsilon_{\text{im}}\;\sim N\left(0,\sigma^{2}\right)$.

#### 2.3.4 Our analysis

Our analysis can be divided into several parts:

1. **Axonal Morphology:** Here, the aim was to assess how morphological features such as undulations and beading impact the SMI estimations. The analysis comprised two parts: variation of undulation strength and variation of beading strength. The axonal water fraction *f* was kept constant at 0.5 in both parts.
  - Undulations (or tortuosity, or sinuosity) were introduced by setting the *ϵ* parameter in CATERPillar to values above zero, ranging from 0.1 to 0.4 across four different substrates. This resulted in undulation coefficients varying between 1.05 and 1.3, which are consistent with values reported for WM tissue (Abdollahzadeh et al., 2021; Stepanyants et al., 2004). All other parameters were kept to their default values (Table 1).
  - Axonal beading (or calibre variation) was introduced by varying the σ parameter in the CATERPillar tool. This parameter ranged from 0 to 0.3 in four different substrates, with higher values producing greater beading strengths. The degree of beading was quantified by the mean COV of the axonal radius along each axon’s length, averaged across all axons within the voxel, which ranged between 0.1 and 0.4. Comparable COV values have been reported in previous electron microscopy studies of WM tissue (H. H. Lee et al., 2019; H.-H. Lee et al., 2024), with typical mean COVs around 0.2–0.3.
2. **Axonal Packing:** The axonal water fraction *f* varied from 0.1 to 0.6 in 6 different substrates.
3. **Orientation Dispersion:** Substrates with a constant *f* of 0.3 were generated while systematically varying the degree of fibre dispersion. All other parameters were assigned their default values (Table 1). In total, five substrate configurations were simulated: (A) three mutually perpendicular fibre populations, (B) two perpendicular populations arranged as planar sheets, (C) two perpendicular and intersecting populations, (D) a single fibre population exhibiting fanning modelled using a Watson distribution, and (E) a single population without fanning. The highest degree of orientation dispersion (i.e., the lowest *p*_2_) was observed in the configuration with three perpendicular populations (A), as this arrangement produced an approximately isotropic voxel.
4. **Cell Permeability:** In this part, we aimed to assess the impact of cell membrane permeability on the SMI results. Permeability was applied to axons and to astrocytes in two separate analyses.
  - We used a substrate with undulating and beaded axons with a packing f=0.5 . The membrane permeability of these axons ranged from 0 (no permeability) to 4$\times \;10^{-5}m\text{/}s$ , as previously reported in experimental studies (Boss et al., 2013).
  - In a substrate containing an axonal packing of 0.5, this time we also included astrocytes. These cells are composed of a soma (representing 1% of the total voxel volume) and of processes (representing 6% of the total voxel volume). The volume fractions for astrocytes were chosen to match those reported in Coelho et al. (2018) for deep WM. The astrocyte membrane permeability ranged from 0 to 10^−4  m/s^ to also match results obtained from Boss et al. (2013).
5. **Myelin:** A substrate was generated consisting of axons surrounded by an outer layer representing the myelin sheath. The intra-axonal volume fraction (AVF) combined with the myelin volume fraction (MVF) was approximately 0.5, with 0.24 corresponding to AVF and 0.26 to the MVF. The myelin thickness for each axon was determined as a function of the axonal radius, according to the relationship described by H. H. Lee et al. (2019). Axonal radii were sampled from a Gamma distribution with parameters α = 2 and β=0.12 , which was different from the default values of α and β so that we this time mimic the distribution of the inner rather than outer-axonal radii. This substrate composed of realistically myelinated axons served as the baseline model. Subsequently, the myelin thickness of all axons was progressively reduced by −25%, −50%, and −75%, as well as a final case with no remaining myelin. These modifications yielded MVFs of 0.263, 0.225, 0.125, 0.075, and 0 respectively.
  It is important to distinguish AVF, AVF+MVF, and the axonal water fraction *f* defined in the SM. Since myelin is considered an MR-invisible compartment, it does not contribute to the total dMRI signal. Consequently, when myelin is present in the substrate, the following relationships hold:
  $$\begin{array}{l} f=\frac{\text{AV}}{\text{AV}+\text{ECV}},\;\;\text{AVF}=\frac{\text{AV}}{\text{AV}+\text{ECV}+\text{MV}},\\ \text{AVF}+\text{MVF}=\frac{\text{AV}+\text{MV}}{\text{AV}+\text{ECV}+\text{MV}}, \end{array}$$ (3)
  and therefore, if MVF≠0 , then f≠AVF≠AVF+ MVF , where AV is the intra-axonal volume, ECV the extracellular volume and MV the myelin volume.
6. **Diffusion Time:** For this analysis, a single numerical substrate (f=0.5 ) was used. To sample signals across various diffusion times, PGSE sequences were applied with a fixed gradient duration (δ=16.5 ms) and varying gradient separations (Δ∈{20,50,75,100} ms). These parameters yielded effective diffusion times (Δ−δ/3 ) of 14.5, 44.5, 69.5, and 94.5 ms. GT diffusivity metrics were extracted directly from the particle trajectories at diffusion times corresponding to the echo time (TE=Δ+δ+5 ms), resulting in TE values of 41.5, 71.5, 96.5, and 121.5 ms. To extend the GT reference data towards shorter diffusion times, additional GT diffusivities were computed at 14.5, 28.5, and 31.5 ms.
7. **Assessing potential confounds:** To evaluate the inherent inter-substrate variability and its potential confounding effect on SMI estimates, we performed a baseline reproducibility analysis. We independently generated four distinct substrates, all adhering to the same target microstructural parameters (using default values shown in Table 1) and featuring non-myelinated axons. The packing density was set to 0.5, and the resulting substrates had GT f values varying from 0.5 to 0.51. Following generation, we simulated the corresponding diffusion-weighted signals for each substrate using the MC simulator and subsequently fit the SMI framework to these signal profiles.
  Furthermore, to rule out other potential confounding effects on the SMI parameter estimations, we evaluated the axon radii distributions across the substrates used in our analysis. These substrates independently varied in axonal water fraction (*f*), orientation dispersion (*p*_2_), undulation, and beading strength.

### 2.4 Estimating GT values

The SMI estimates obtained in each configuration were compared to their corresponding effective GT values. These GT values were obtained for *f*, *p*_2_ and diffusivities as follows:

1. **GT AVF:** To estimate the total AVF, we modelled the segments between consecutive points along the axonal skeletons as truncated cones. Using the 3D coordinates and radii of the skeleton nodes, the volume *V* of each segment was calculated as:
  $$V=\frac{1}{3}\pi h\left(R^{2}\;+Rr+r^{2}\right)$$ (4)
  where *R* and *r* denote the radii of two adjacent spherical nodes, and *h* is the Euclidean distance between their centres. These segmental volumes were summed across all axons and subsequently divided by the total voxel volume to yield the GT AVF. To prevent boundary artifacts, nodes extending beyond the voxel edge by more than half of their radius were excluded from this calculation.
2. **GT fODF:** We selected point pairs separated by a fixed arc-length from 3D points within each axonal skeleton. This arc-length was set to 15 μm so that it would be approximately equal to the diffusion length of intracellular water particles, assuming a free IAS diffusivity $D_{0}\;=2.5\mu {\text{m}}^{2}\text{/ms}$ and a diffusion time of 50 ms. For each pair, a unit directional vector was computed. Vectors were aggregated across all axons into a single global orientation tensor. Following normalization, the tensor’s principal eigenvalue (*c*_2_) was extracted via eigendecomposition to quantify the dominant alignment variance. The final orientation dispersion parameter was then derived using $p_{2}\;=1.5c_{2}\;-0.5$ , resulting in a value between 0 (isotropic) and 1.0 (perfectly parallel and straight axons). In substrates exhibiting some degree of axonal undulations, the effective GT *p*_2_ values were therefore below 1 even when the axons were overall parallel. Indeed, because our chosen arc-length reflects the diffusion coarse-graining distance but is relatively short compared to total axonal length, GT *p*_2_ is sensitive to local axonal dispersions as well as to tract-level crossings that happen on longer length scales.
3. **GT diffusivities:** The GT diffusivity metrics *D_e_*_⊥_, *D_e_*_∥_, and *D_a_* were derived directly from the water molecule trajectories generated by the MC simulations. These diffusivities were estimated using the standard Einstein equation for diffusion:
  $$D=\frac{1}{2dt}\left(\frac{1}{N}\sum_{i=1}^{N}\Delta \;x_{i}^{2}\right)$$ (5)
  where *D* represents the respective diffusivity, *d* denotes the relevant spatial dimensionality (*d* = 1 for axial diffusion and *d* = 2 for radial diffusion), *t* is the total diffusion time, and the term in parentheses is the mean squared displacement (MSD) averaged over *N* walkers. To approximate the MSD along the axis of interest, the net displacement Δ*x_i_* for each walker was computed as the difference between its final and initial spatial coordinates. The total duration of the diffusion process in this case is TE = Δ+δ+5 ms, which incorporates a 5 ms temporal padding prior to sequence initiation. Accordingly, GT *D_e_*_⊥_ and *D_e_*_∥_ were calculated from the radial and axial MSDs of the extracellular walkers, respectively, while GT *D_a_* was computed from the axial MSD of the intracellular walkers.

In the case of permeable cells, the water molecules from different compartments could not be separated, so the GT compartment-specific diffusivity could not be obtained. In these cases, we instead computed *D*_⊥_ and *D*_∥_ which indicated the perpendicular and parallel diffusivity of both compartments combined.

## 3 Results

The following subsections present the effects of specific parameters on the SMI parameter estimates. Each parameter was varied independently while all others were kept constant. Figures 4–11 illustrate the influence of each parameter on the SMI estimates, both with and without the addition of Gaussian noise. Comparable results using Rician noise are provided in Supplementary Figures S5–S12. Figures 12 and 13 summarise the overall impact of all parameters on the SMI estimates, and the error to their corresponding GT in the absence of noise, respectively.

### 3.1 Axonal undulations

As previously described, axonal undulation was quantified as the ratio of true axonal length to the straight-line distance between endpoints. As shown in Figure 4, increasing the undulation coefficient led to decreased SMI estimates for *p*_2_ (-0.124), *D_a_* (-0.216 $\mu {\text{m}}^{2}\text{/ms}$ ), and *D_e_*_∥_ (-0.156 $\mu {\text{m}}^{2}\text{/ms}$ ). Conversely, the perpendicular extra-axonal diffusivity (*D_e_*_⊥_) increased by 0.105 $\mu {\text{m}}^{2}\text{/ms}$ . The estimated *f* remained highly stable, showing a negligible increase of 0.006. The estimation errors remained broadly consistent across all levels of undulation, with the notable exceptions of *D_a_* and *p*_2_. At maximum undulation, the absolute overestimation for *D_a_* increased from 0.13 to 0.23 $\mu {\text{m}}^{2}\text{/ms}$ , while the underestimation for *p*_2_ rose from 0.12 to 0.16.

**Fig. 4.**
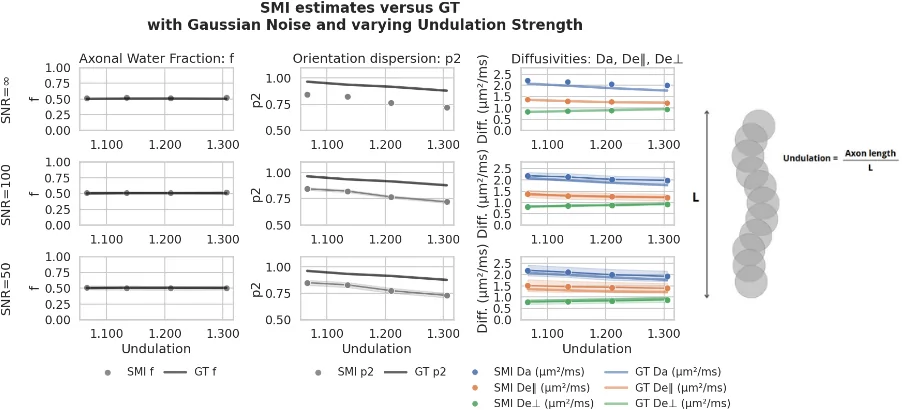
SMI estimates of *f*, *p*_2_, *D_e_*_∥_, *D_e_*_⊥_, and *D_a_* are shown together with their corresponding GT values as a function of the mean undulation coefficient for each voxel. When noise is included, the shaded areas represent the standard deviation across 1000 repetitions.

### 3.2 Axonal beading

Beading was modulated across substrates by varying the parameter σ (Fig. 2) from 0 to 0.3. The degree of beading was quantified as the mean COV of the axonal radius along each axon’s length, averaged across all axons within the voxel. As shown in Figure 5, increasing the mean COV from 0.1 to 0.4 led to absolute increases in the estimated *f* (+0.121) and *D_e_*_⊥_ (+0.042 $\mu {\text{m}}^{2}\text{/ms}$ ), while *p*_2_ (-0.210), *D_a_* (-0.412 $\mu {\text{m}}^{2}\text{/ms}$ ), and *D_e_*_∥_ (-0.035 $\mu {\text{m}}^{2}\text{/ms}$ ) decreased. The absolute errors of these estimates with respect to the effective GT were more strongly influenced by beading for *f*, *p*_2_, and *D_a_* than for the extra-axonal diffusivities. Specifically, higher beading amplitudes caused the estimation errors to grow substantially for *f* (from 0.00 to 0.09), *p*_2_ (from 0.09 to 0.27), and *D_a_* (from 0.18 to 0.28 $\mu {\text{m}}^{2}\text{/ms}$ ), whereas the errors for *D_e_*_∥_ and *D_e_*_⊥_ remained highly stable, varying by only -0.022 and +0.015 $\mu {\text{m}}^{2}\text{/ms}$ , respectively.

**Fig. 5.**
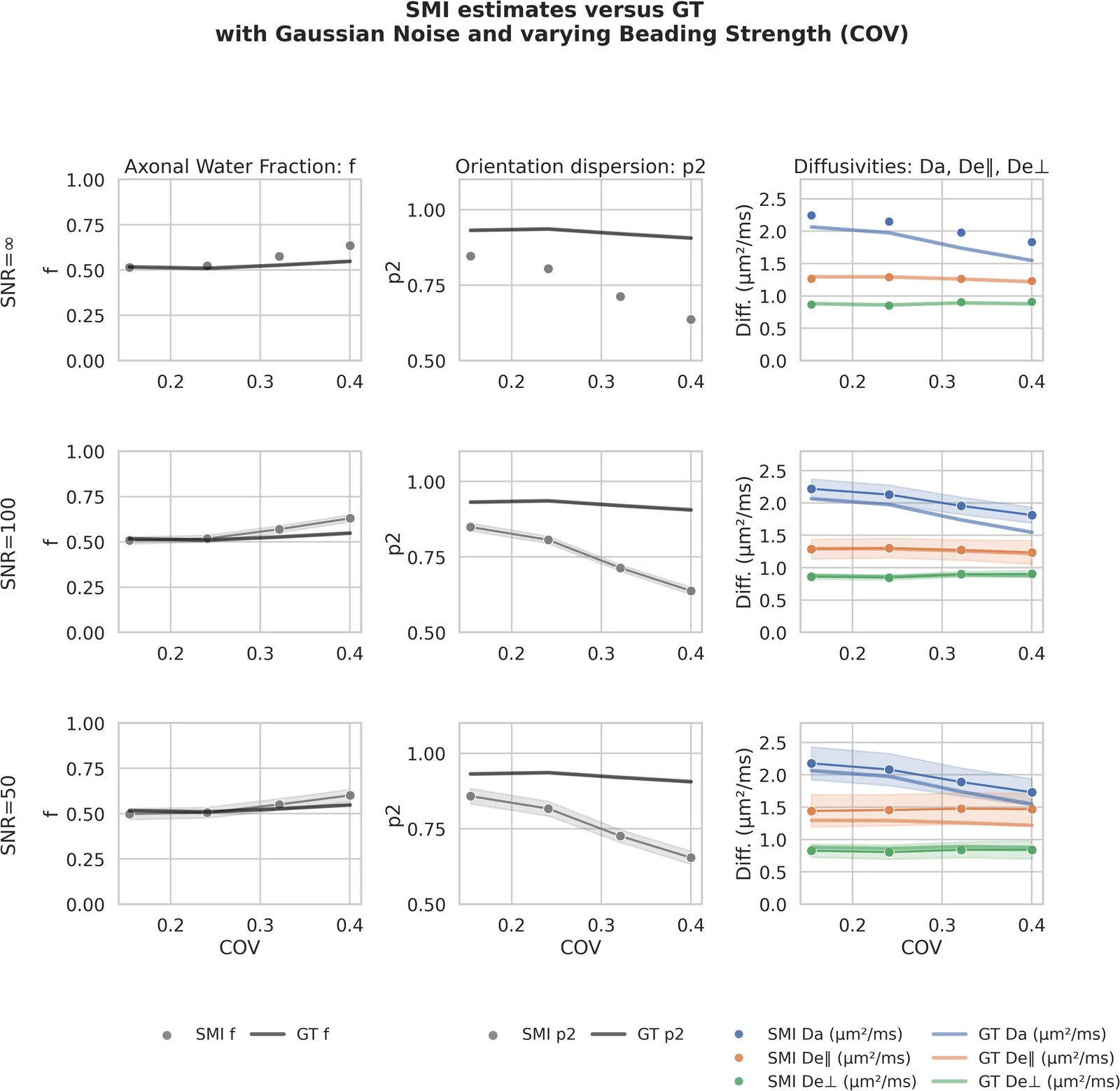
SMI estimates of *f*, *p*_2_, *D_e_*_∥_, *D_e_*_⊥_, and *D_a_* are shown together with their corresponding GT values as a function of the mean COV for each voxel. This COV quantified the beading strength of the axons, which was modulated across subjects by varying the parameter σ from 0 to 0.3. The mean COV of the axonal radius was obtained by averaging the COV for all axons within the same voxel. When noise is included, the shaded areas represent the standard deviation across 1000 repetitions.

### 3.3 Axonal packing density

In Figure 6, the SMI estimates varied with increasing GT *f*. The estimated *f* increased by 0.511 (from 0.11 to 0.62) over the evaluated range, with a negligible absolute error that remained between 0.00 and 0.02. The estimates of *p*_2_ displayed a bell-shaped evolution, yielding a net increase of 0.166, while its absolute estimation error improved from 0.35 at the lowest packing density down to 0.19 at the highest. *D_a_* also displayed a mild bell-shaped pattern with increasing GT *f*, resulting in a net change of -0.078 $\mu {\text{m}}^{2}\text{/ms}$ , while its absolute error grew from 0.04 to 0.13 $\mu {\text{m}}^{2}\text{/ms}$ . As expected, all EAS diffusivity estimates decreased with higher packing densities: *D_e_*_∥_ dropped by 0.292 $\mu {\text{m}}^{2}\text{/ms}$ and *D_e_*_⊥_ dropped by 0.567 $\mu {\text{m}}^{2}\text{/ms}$ . Their respective absolute errors remained relatively stable, showing slight improvements by decreasing 0.036 and 0.055 $\mu {\text{m}}^{2}\text{/ms}$ over the entire range.

**Fig. 6.**
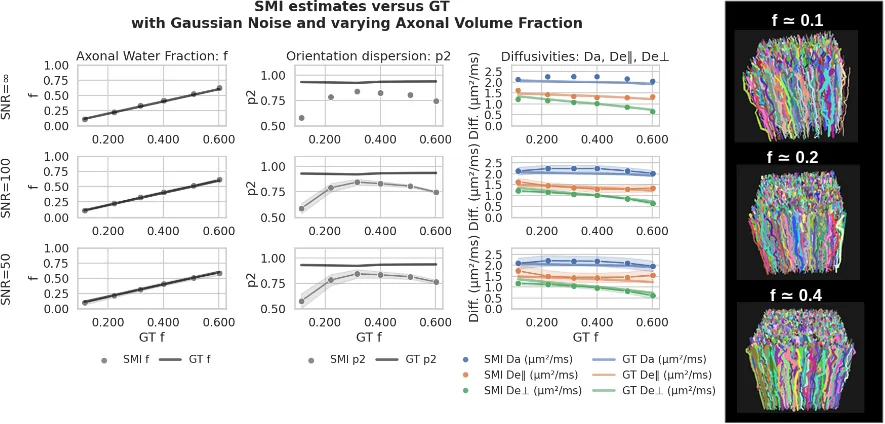
SMI estimates of *f*, *p*_2_, *D_e_*_∥_, *D_e_*_⊥_, and *D_a_* are shown together with their corresponding GT values as a function of the GT axonal water fraction *f*. Example of substrates with various values of f are shown. When noise is included, the shaded areas represent the standard deviation across 1000 repetitions. With increasing f, the COV for beading strength increased from 0.21 at the lowest packing to 0.28, while the undulation factor concurrently rose from 1.10 to 1.145.

### 3.4 Orientation dispersion

Orientation dispersion, represented by the GT *p*_2_, was varied by including different numbers of fibre populations and by inducing axonal fanning. Substrate (A) contained three perpendicular fibre populations, while substrates (B) and (C) each included two perpendicular populations arranged in sheet-like and interwoven configurations, respectively. Substrate (D) consisted of a single fibre population with fanning, modelled using a Watson distribution, and substrate (E) featured a single population with no fanning. As shown in Figure 7B and C revealed very similar parameter estimates, as they were both composed of two perpendicular fibre populations. This similarity suggests that the SMI is not sensitive to different crossing configurations. The run-times necessary for each substrate are illustrated in Supplementary Figure S2.

**Fig. 7.**
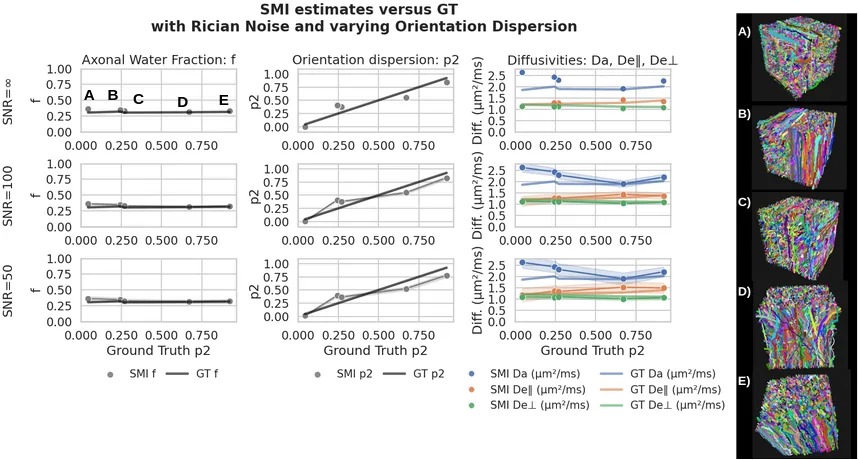
SMI estimates of *f*, *p*_2_, *D_e_*_∥_, *D_e_*_⊥_, and *D_a_* are shown together with their corresponding GT values as a function of the mean GT *p*_2_ for each voxel. Substrate (A) contained three perpendicular fibre populations, while substrates (B) and (C) each included two perpendicular populations arranged in sheet-like and interwoven configurations, respectively. Substrate (D) consisted of a single fibre population with fanning, modelled using a Watson distribution, and substrate (E) featured a single population with no fanning. By comparing the SMI parameters from the varying-fODF substrates against the substrate (E), we can disentangle the influence of macroscopic orientation dispersion from microscopic morphological changes. When noise is included, the shaded areas represent the standard deviation across 1000 repetitions.

The estimated *p*_2_ followed an increasing trend, yielding a net absolute increase of 0.841 across the range, while its absolute estimation error varied from 0.04 to 0.08. With increasing *p*_2_ (corresponding to greater axonal alignment), the estimated *f* decreased slightly by 0.032 (from 0.36 to 0.33). *D_e_*_⊥_ and *D_a_* decreased by 0.050 $\mu {\text{m}}^{2}\text{/ms}$ (from 1.12 to 1.07) and 0.377 $\mu {\text{m}}^{2}\text{/ms}$ (from 2.63 to 2.25) respectively. Conversely, *D_e_*_∥_ increased by 0.212 $\mu {\text{m}}^{2}\text{/ms}$ (from 1.13 to 1.34) across the full range. These estimates stayed broadly in line with their effective GT values. The absolute errors of *D_e_*_⊥_ and *D_e_*_∥_ remained exceptionally low and stable, improving by 0.056 and 0.027 $\mu {\text{m}}^{2}\text{/ms}$ , respectively. However, at low GT *p*_2_ values (A–C), *D_a_* was severely overestimated, showing an initial absolute error of 0.77 $\mu {\text{m}}^{2}\text{/ms}$ . This overestimation dropped substantially by 0.539 $\mu {\text{m}}^{2}\text{/ms}$ as alignment increased, yielding a much lower final estimation error of 0.23 $\mu {\text{m}}^{2}\text{/ms}$ for the perfectly aligned substrate.

### 3.5 Axonal membrane permeability

In Figure 8, the membrane permeability of all axons was increased from 0 (impermeable) to $4\times 10^{-5}\text{ m/s}$ in steps of $1\times 10^{-5}\text{m/s}$ . This led to a substantial absolute decrease of 0.410 in the estimated *f* (dropping from 0.54 to 0.13), despite its GT remaining constant. This resulted in the absolute estimation error for *f* surging from 0.03 up to 0.38 at the maximum permeability of $4\times 10^{-5}\text{ m/s}$ . In contrast, *p*_2_ and *D_a_* remained relatively stable, increasing by only 0.048 (from 0.80 to 0.85) and 0.1 $\mu {\text{m}}^{2}\text{/ms}$ (from 2.14 to 2.24 $\mu {\text{m}}^{2}\text{/ms}$ ), respectively, with the absolute error of *p*_2_ slightly improving. The extracellular diffusivities *D_e_*_∥_ and *D_e_*_⊥_ varied more substantially, with *D_e_*_∥_ increasing by 0.491 $\mu {\text{m}}^{2}\text{/ms}$ (from 1.29 to 1.78 $\mu {\text{m}}^{2}\text{/ms}$ ) and *D_e_*_⊥_ decreasing by 0.367 $\mu {\text{m}}^{2}\text{/ms}$ (from 0.85 to 0.48 $\mu {\text{m}}^{2}\text{/ms}$ ).

**Fig. 8.**
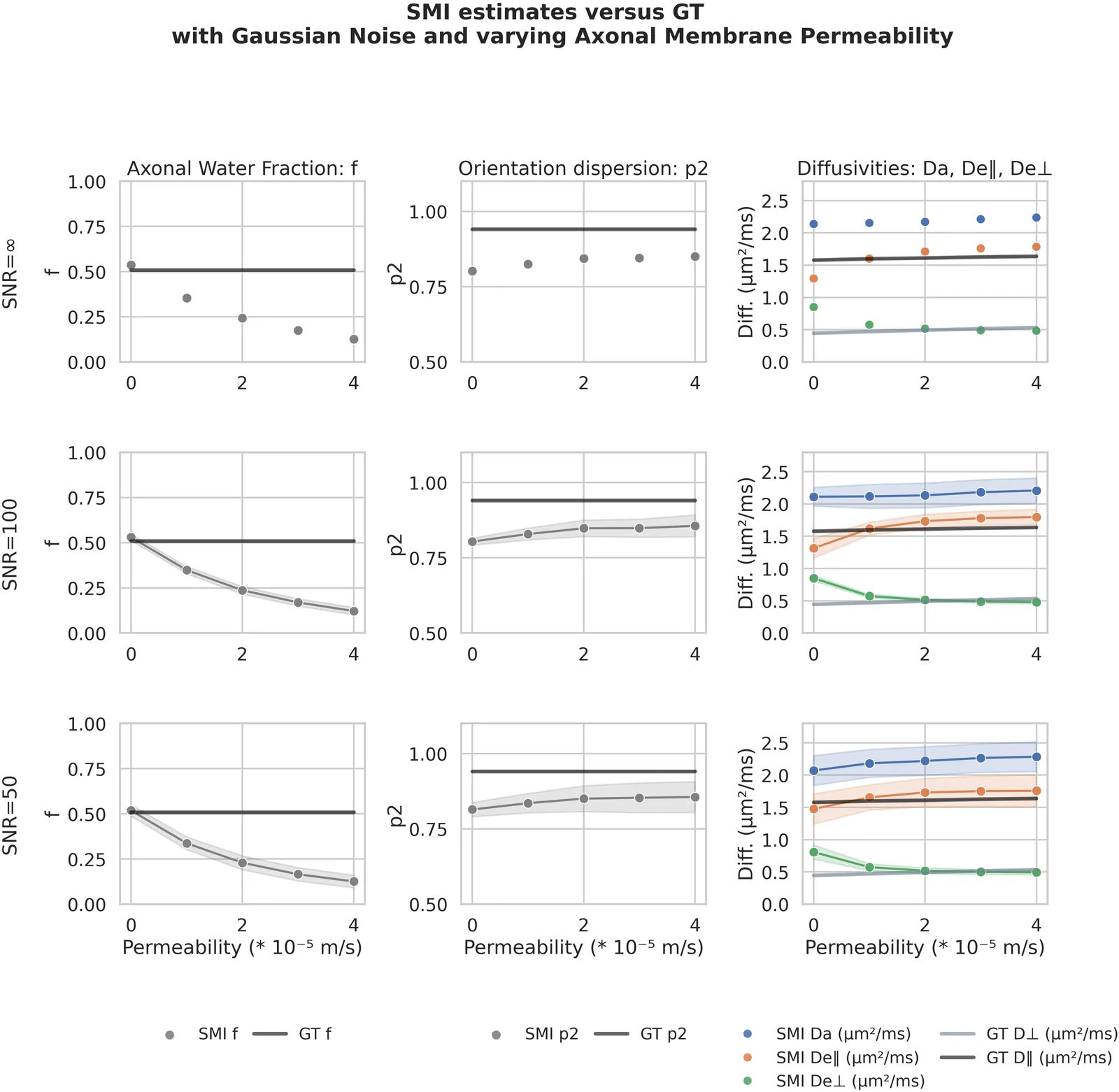
SMI estimates of *f*, *p*_2_, *D_e_*_∥_, *D_e_*_⊥_, and *D_a_* are shown together with their corresponding GT values as a function of the membrane permeability of the axons for each voxel. When noise is included, the shaded areas represent the standard deviation across 1000 repetitions.

A direct comparison between the diffusion estimates and their GTs was not possible, as the presence of permeability prevented clear separation of intracellular and extracellular trajectories. Instead, the GT *D*_⊥_ and *D*_∥_, which are not compartment-specific, were reported and both increased slightly with axonal membrane permeability. At zero permeability, *D*_⊥_ was positioned between *D_e_*_⊥_ and $D_{a⊥}=0$ , while *D*_∥_ was positioned between *D_e_*_∥_ and *D_a_*.

### 3.6 Astrocyte membrane permeability

Astrocytes were included in voxels containing axons, with volume fractions and morphological parameters adjusted to match realistic astrocyte properties in deep WM, as described previously (Coelho et al., 2018). The membrane permeability of astrocytes was varied from 0 (impermeable) to $10^{-4}\text{ m/s}$ . In Figure 9, AVF denotes the axonal volume fraction, while AVF + AsVF represents the combined volume fraction of axons and astrocytes (processes and soma). Astrocytes had the greatest influence on the SMI estimates at low permeability. Increasing astrocyte membrane permeability improved the axonal estimation accuracy of both *f* and *p*_2_. At zero permeability, *f* was overestimated with an absolute error of 0.11 and this error dropped to 0.03 at maximum permeability. For *p*_2_, the absolute estimation error improved from 0.22 down to 0.1. The diffusivity estimates were largely stable, showing only minor changes across the full permeability range: *D_a_* increased by 0.071 $\mu {\text{m}}^{2}\text{/ms}$ , while the EAS diffusivities slightly decreased, with *D_e_*_∥_ dropping by 0.070 $\mu {\text{m}}^{2}\text{/ms}$ and *D_e_*_⊥_ dropping by 0.031 $\mu {\text{m}}^{2}\text{/ms}$ . GT values of *D*_∥_ and *D*_⊥_, which are not specific to a compartment, both increased by approximately 0.03 $\mu {\text{m}}^{2}\text{/ms}$ across the full range of astrocyte membrane permeability.

**Fig. 9.**
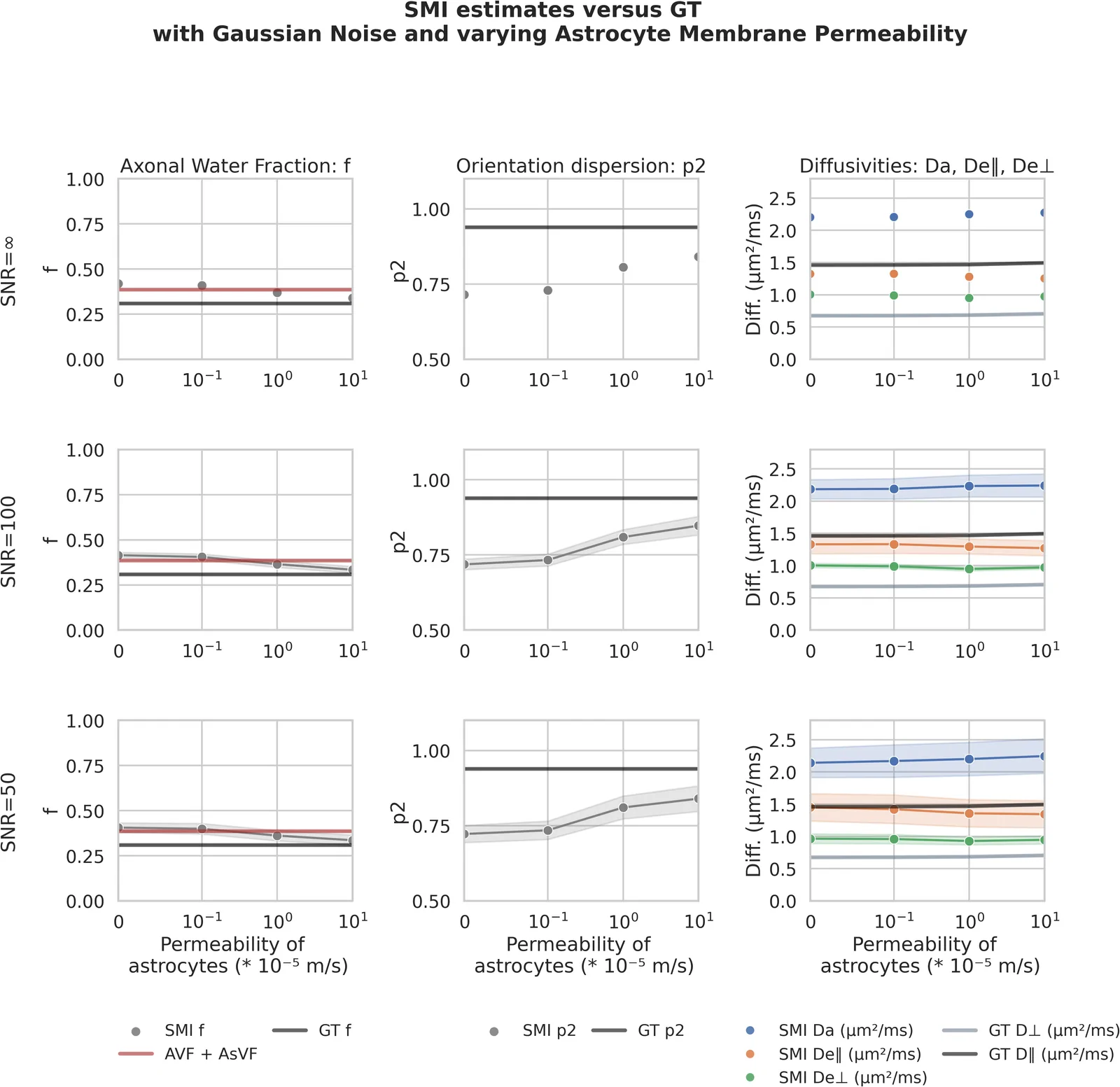
SMI estimates of *f*, *p*_2_, *D_e_*_∥_, *D_e_*_⊥_, and *D_a_* are shown together with their corresponding GT values as a function of the membrane permeability of the astrocytes for each voxel. The astrocytes’ soma and processes took 1% and 6% of the total voxel volume. Additionally to GT *f*, the SMI *f* can be compared to AVF + AsVF, which accounts for the volume occupied by both axons and astrocytes. When noise is included, the shaded areas represent the standard deviation across 1000 repetitions.

### 3.7 Myelin volume fraction

Figure 10 illustrates the behavior of the estimates when a myelin sheath was introduced and progressively thickened until reaching a realistic g-ratio, as described by H. H. Lee et al. (2019). The estimated *f* increased by an absolute value of 0.158 across the full range, remaining positioned midway between AVF+MVF and AVF. It became increasingly overestimated relative to its GT, with the absolute estimation error growing from 0.01 to 0.17 at the highest MVF. The parameter *p*_2_ remained highly stable across varying MVF values (changing by only -0.002, effectively remaining at 0.73), with a consistent absolute estimation error of 0.22 throughout the range. In contrast, *D_a_* was systematically overestimated and further increased slightly by 0.019 $\mu {\text{m}}^{2}\text{/ms}$ despite a constant GT. The EAS diffusivities both decreased with increasing MVF: *De*_∥_ decreased slightly by 0.053 $\mu {\text{m}}^{2}\text{/ms}$ , while *D_e_*_⊥_ decreased dramatically by 0.423 $\mu {\text{m}}^{2}\text{/ms}$ . Their absolute errors remained very low, improving slightly down to 0.02 and 0.01 $\mu {\text{m}}^{2}\text{/ms}$ respectively, at maximum permeability.

**Fig. 10.**
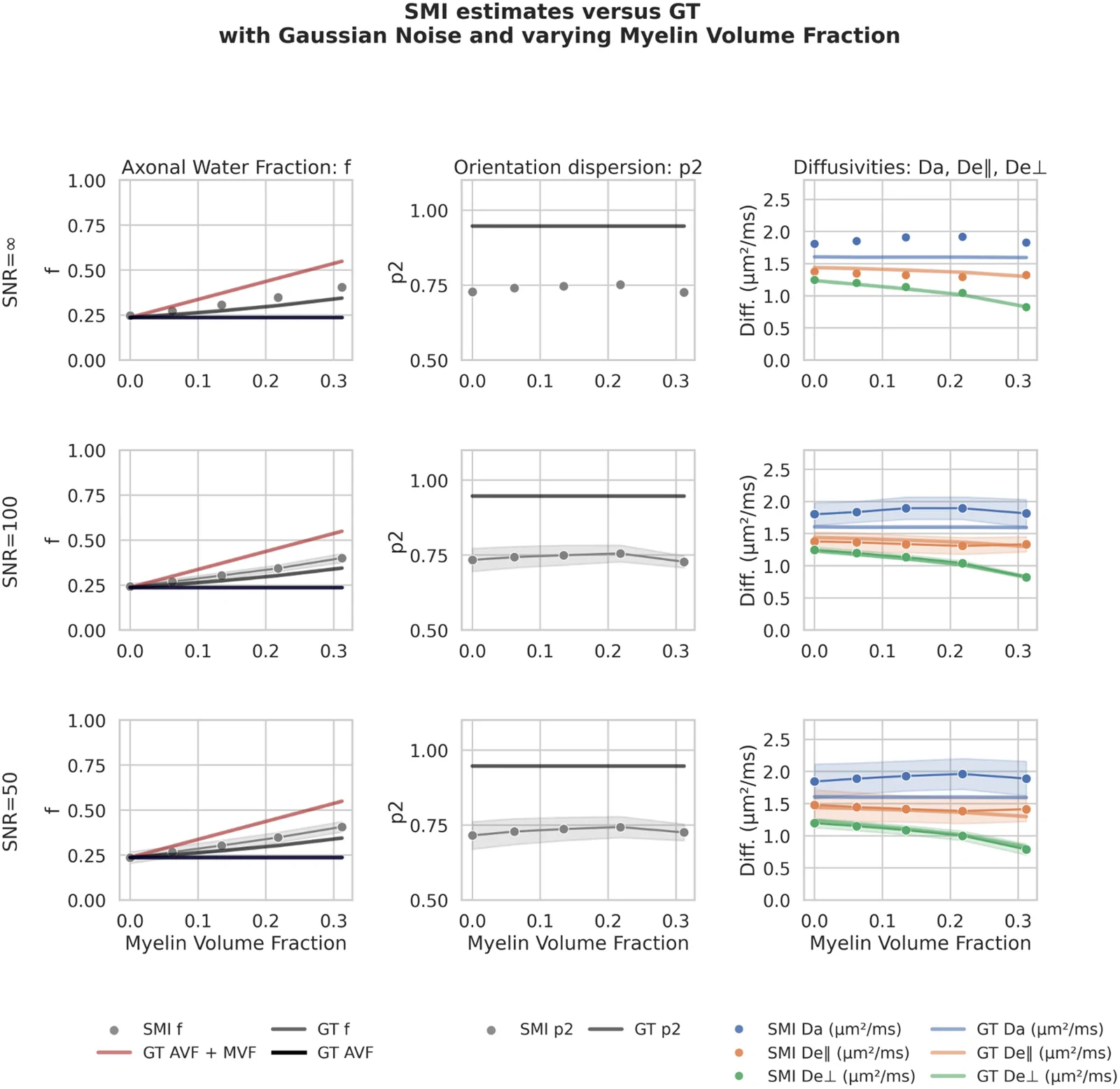
SMI estimates of *f*, *p*_2_, *D_e_*_∥_, *D_e_*_⊥_, and *D_a_* are shown together with their corresponding GT values as a function of the MVF for each voxel. GT f takes into account the reduced water volume with increasing MVF, thus it increases despite a constant AVF. The AVF + MVF represents the real volume fraction of axons with myelin included. The estimated *p*_2_ is particularly low due to the low GT AVF. When noise is included, the shaded areas represent the standard deviation across 1000 repetitions.

### 3.8 Diffusion time

To investigate the effect of diffusion time, a fixed substrate containing both undulating and beading axons was evaluated at a constant packing density of 0.5. Figure 11 compares the resulting SMI parameter estimates against their respective GT. A mismatch in the list of evaluated diffusion times between the SMI estimates and the GT was present due to the different definitions of diffusion times as mentioned in Section 2.3.4.

**Fig. 11.**
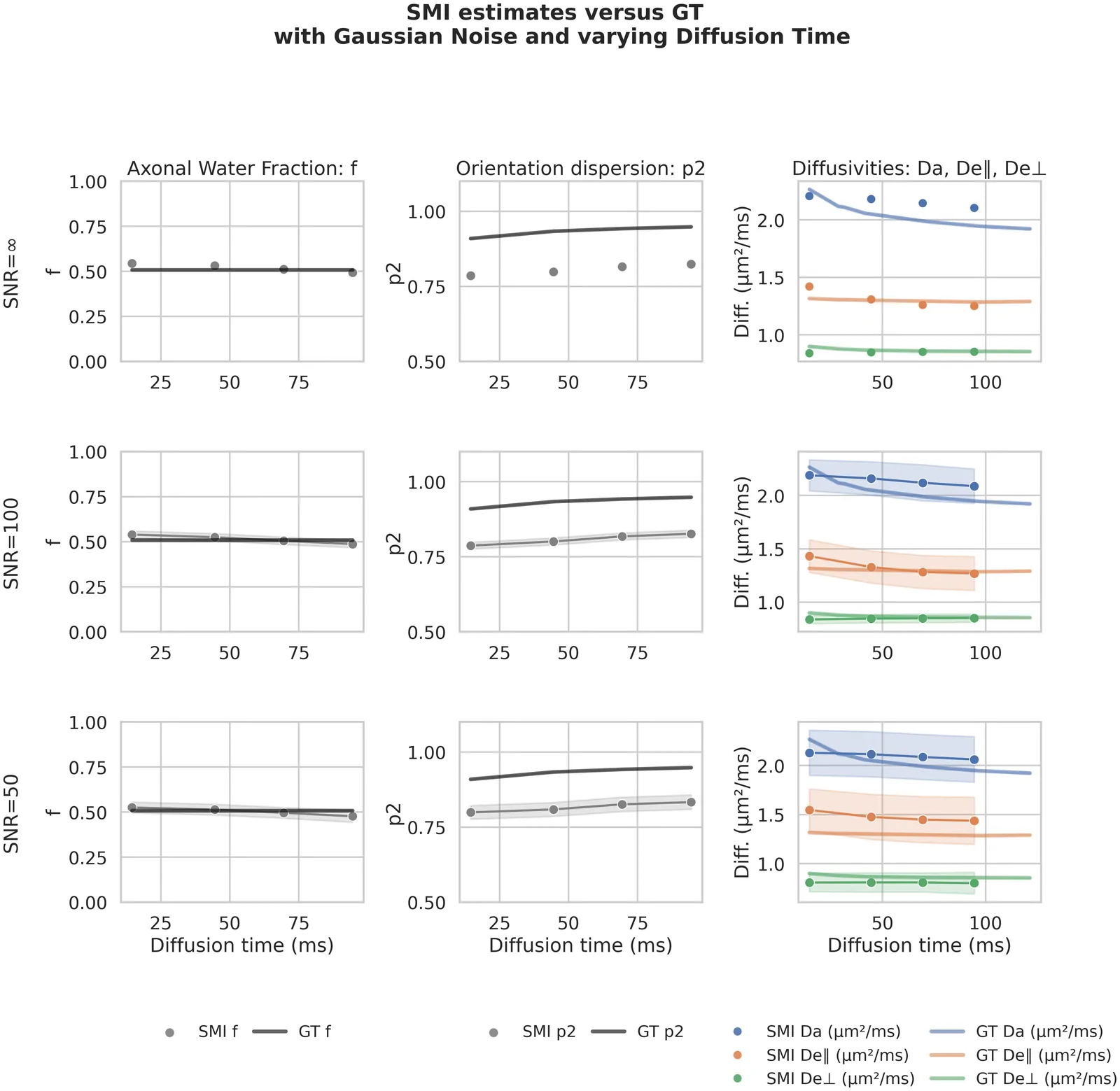
SMI estimates of *f*, *p*_2_, *D_e_*_∥_, *D_e_*_⊥_, and *D_a_* are shown together with their corresponding GT values as a function of the diffusion time for each voxel. The diffusion time for SMI estimates and GT *p*_2_ was defined as $\Delta -\frac{\delta }{3}$ whereas for the GT diffusivities, it was defined by TE though $TE>\Delta -\frac{\delta }{3}$. When noise is included, the shaded areas represent the standard deviation across 1000 repetitions.

All SMI estimates demonstrated some sensitivity to the diffusion time. Specifically, a slight decrease was observed for *f* (-0.051), *D_a_* (-0.103  $\mu {\text{m}}^{2}\text{/ms}$ ), and *D_e_*_∥_ (-0.169  $\mu {\text{m}}^{2}\text{/ms}$ ) across the range 14.5–94.5 ms. Conversely, *p*_2_ and *D_e_*_⊥_ exhibited an increase of 0.039 and 0.013 $\mu {\text{m}}^{2}\text{/ms}$ , respectively. As expected, all GT diffusivities exhibited a consistent downward trend over time, reflecting their physical convergence towards the long-time limit (*D*_∞_). When comparing the model estimates to these GT values, estimation accuracy proved highly dependent on the diffusion time. At times exceeding 20 ms, the EAS diffusivities were estimated accurately, whereas *D_a_* was systematically overestimated. Conversely, at the shortest diffusion time evaluated (t=14.5 ms), the EAS estimates became slightly inaccurate, resulting in an overestimation of *D_e_*_∥_ and an underestimation of *D_e_*_⊥_.

### 3.9 Noise

In Figures 4–11, the addition of Gaussian noise produced similar effects across most simulations. As the SNR decreased (i.e., as noise levels increased), the standard deviation widened accordingly. In addition, noise introduced a systematic bias as an overestimation of *D_e_*_∥_. This effect was particularly pronounced at an SNR of 50. When simulating Rician noise instead, the results for every part of the analysis were very similar, and can be found in Supplementary Figures S5–S12.

### 3.10 Assessment of confounds

As illustrated in Supplementary Figure S3, the derived SMI parameter estimates as well as GT values remained highly consistent across identically parametrised substrates. This high degree of consistency demonstrates that the SMI model is robust to the random geometric fluctuations introduced during substrate generation, confirming that estimate changes observed in subsequent experiments are very likely driven by true microstructural variations rather than stochastic effects.

Regarding the radii distributions within substrates used in this work in Supplementary Figure S4, they were highly consistent across all configurations, displaying a mode at approximately 0.5 μm and a tail extending to approximately 6 μm. This structural uniformity confirms that variations in axon calibre did not act as a confounding variable in our SMI estimates.

## 4 Discussion

In this work, the goal was to assess the impact of realistic axon morphology and irregularities (that depart from a stick geometry) on SMI estimates. For this, we evaluated the performance of the SM using numerical substrates of realistic WM generated with CATERPillar. By fitting SMI to signals derived from these substrates, we gained insights into the mechanisms underlying changes in its estimates, as well as the conditions under which its estimations deviated from the GT.

### 4.1 Complex morphologies

In the SM, axons are idealised sticks. The validity of this assumption had been verified in the human brain using very high diffusion weighting, which, indeed, yielded a dependence of the powder-average signal as $1/\sqrt{(}b)$ (Veraart et al., 2019), in agreement with Callaghan’s model of randomly oriented sticks (P. Callaghan et al., 1979). Nonetheless, it has not been established to what extent the effective GT diffusivities vary with morphological complexities such as undulations and beading, nor how biased SMI estimates would be relative to the effective GT. Indeed, our results confirmed the sensitivity of SMI estimates to undulation and beading. Across all tested strengths of beading and undulation, *D_a_* was consistently overestimated while *p*_2_ was underestimated, indicating a systematic bias in the SMI estimates.

Increasing axonal undulations resulted in a marked decrease in the effective GT values for *p*_2_, *D_a_*, and *D_e_*_∥_. The orientation dispersion reflected by *p*_2_ was derived from water diffusion over 50 ms, corresponding to a diffusion length of about 15 μm. Because undulations can occur over such short length scales, both micro- and macroscopic orientation dispersion dictate the total orientation dispersion (Brabec et al., 2020), therefore higher undulation amplitudes predictably reduced the effective *p*_2_. Similarly, effective *D_a_* and *D_e_*_∥_ decreased because undulations mechanically hindered diffusion along the primary axonal axis. Conversely, an increase was observed in effective *D_e_*_⊥_, which likely arises from the formation of larger inter-axonal gaps oriented perpendicular to the fibre direction. Overall, although undulations influenced the effective GT values, the accuracy of the SMI estimates with respect to them remained highly stable, with the exception of *D_a_* which was mildly overestimated at high undulation coefficients.

Increasing the beading strength beyond COV = 0.25 led to a decrease in the GT values of *p*_2_ and *D_a_* (a trend similar to, but more pronounced than the effect of increasing axonal undulation) alongside an increase in *f*. While the overall trends of the SMI estimates tracked those of their corresponding GTs, the magnitude of the estimation errors worsened at higher beading amplitudes, resulting in progressive overestimations of *f* and *D_a_*, and a progressive underestimation of *p*_2_. Interestingly, the diffusion signal for all beaded substrates consistently exhibited a $1/\sqrt{b}$ decay, entirely independent of the beading strength. This suggests that the observed estimation biases were not caused by a violation of the high-*b* power-law assumption.

In healthy WM, the mean COV of axonal radius is typically around 0.2–0.3, as reported by H. H. Lee et al. (2019), H.-H. Lee et al. (2024), and Tian et al. (2025). At this beading level, the inaccuracies of these parameters were rather mild, indicating that *f*, *p*_2_, and *D_a_* may not be too biased by beading under normal conditions. In contrast, as increased axonal beading has been reported in pathological tissues, the resulting overestimation of *f* and *D_a_*, and underestimation of *p*_2_, may be more problematic. Heavily beaded axons may, indeed, no longer be modelled as infinitely long sticks. The beading in pathology could go beyond the range investigated in this study, and thus introduce significant biases when interpreting microstructural alterations using the SM in these contexts.

The sensitivity of dMRI signals to axonal beading and undulations has been examined in several previous studies employing MC simulations in numerical substrates that were: created from scratch (Brabec et al., 2020; Budde & Frank, 2010; Nilsson et al., 2011), segmented from EM (Andersson et al., 2020; H. H. Lee et al., 2019, 2020; H.-H. Lee et al., 2024) and segmented from x-ray images (Andersson et al., 2020, 2022). Some of these studies (Budde & Frank, 2010; Nilsson et al., 2011) reported decreases in mean diffusivity (MD) and/or apparent diffusion coefficient (ADC) with increasing undulation amplitude and beading strength. Further experimental investigations have confirmed that such morphological alterations occur due to axonal damage in pathological conditions such as spinal cord injury (Loy et al., 2007), multiple sclerosis (Budde et al., 2008; Luchicchi et al., 2021; Pavan, Alemán-Gómez, Jenni, et al., 2025), traumatic brain injury (Johnson et al., 2013) and ischemic stroke (Budde & Frank, 2010), where reductions in parallel diffusivity have been proposed as a biomarker of axonal damage (Budde & Frank, 2010; Fieremans et al., 2013; Hui et al., 2012; H.-H. Lee et al., 2024; Loy et al., 2007; Pavan, Alemán-Gómez, Jenni, et al., 2025). Our findings are, therefore, in strong agreement with these previous results, as both *D_a_* and *D_e_*∥ (particularly *D_a_*) exhibited substantial decreases with increasing undulation and beading strength.

### 4.2 Axonal packing density

In this section, the substrates contained axons that were both beaded and undulating, with varying packing densities.

The accuracy and precision on *f* estimates were excellent throughout the range considered. Surprisingly though, our results revealed that for GT *f* values below 0.3, the underestimation of *p*_2_ was stronger. According to Cottaar et al. (2020), a more isotropic diffusion signal can arise either from an increase in fibre dispersion (lower *p*_2_) or from reduced anisotropy of the single-fibre response function (for example, due to low packing density or weaker diffusion restriction). It appears that, at low packing density, the model explained the increased isotropy primarily through greater fibre dispersion, leading to underestimated *p*_2_. This is likely due to the model design, where coherent fascicles made up of IAS and proximal EAS were convolved with the ODF. At low *f*, the EAS becomes much looser (*D_e_*_∥_ and *D_e_*_⊥_ nearly coincide) while each axon remains highly anisotropic, thus the medium may be interpreted as highly dispersed. It is worth noting that the lowest packing density (10%) modelled may be unrealistic, as packing densities have been shown to typically range from 60 to 80% in WM (Coelho et al., 2022; Stikov et al., 2015; Syková & Nicholson, 2008).

An increase in the underestimation of *p*_2_ was also observed at GT *f* values above 0.5, resulting in a bell-shaped response curve for the estimated *p*_2_. Subsequent investigation revealed that the COV for beading strength increased from 0.21 at the lowest packing to 0.28 at the highest packing, while the undulation factor concurrently rose from 1.10 to 1.145. Given our prior observation that beading inherently biases the estimated *p*_2_ downwards relative to the GT, it is probable that the *p*_2_ underestimation observed at high packing densities is primarily driven by these emergent beading effects.

The beading and undulation at high packing densities that drove the *p*_2_ underestimation also influenced the estimated *D_a_*. The latter indeed displayed a bell-shaped dependence on fibre packing similar to that of *p*_2_. However, for *D_a_*, the lowest and highest packing densities yielded the most accurate estimates, whereas intermediate packing led to an overestimation. In terms of the effective GT, *D_a_* remained relatively stable across the substrates but exhibited a slight decrease at higher axonal densities.

### 4.3 Orientation dispersion

When assessing the impact of orientation dispersion on parameter estimation, low GT *p*_2_ values were associated with some overestimation of *f* and of *D_a_*, but estimates remained otherwise accurate.

Regarding *D_a_*, estimation accuracy appeared to depend on the underlying fibre configuration. In single-population voxels (D, E), *D_a_* was overestimated for highly aligned axons (substrate E) but accurately estimated when some fanning was present (substrate D). This trend aligns with the negative relationship between *D_a_* and the broadness of the orientation dispersion that was reported when comparing SMI estimates to electron-microscopy data (Coronado-Leija, Abdollahzadeh, et al., 2024). The latter also reported a decreased *D_e_*_∥_ and increased *D_e_*_⊥_ with fibre dispersion, which agrees with our findings. In configurations featuring multiple fibre populations (substrates A–C), the overestimation of *D_a_* became even more pronounced. Because the SM assumes a single fibre population per voxel, complex architectures featuring crossing or diverging fibres are expected to induce larger estimation errors. Consequently, metrics derived from such voxels should be interpreted with caution (A. F. Howard et al., 2022).

Furthermore, when specifically examining voxels with two fibre populations (substrates B and C), *p*_2_ was overestimated. It is somewhat surprising that this effect was not also observed for three fibre populations in substrate A. One possible explanation is that, despite using isotropically distributed encoding directions, the orientations of the two fibre populations were not sufficiently sampled to ensure accurate estimation. Increasing both the number of diffusion-encoding directions (A. Howard et al., 2019) and the number of b-shells (beyond the five used in this study) (Lampinen et al., 2020) may improve the accuracy of SMI parameter estimation.

### 4.4 Axonal membrane permeability

When varying axonal membrane permeability, the progressive underestimation of *f* with increasing membrane permeability aligned with previous studies highlighting the impact of exchange on SM estimates in GM (Jelescu et al., 2022) and tumors (Gardier et al., 2023). The increase of *D_e_*_∥_ and *D_a_* with membrane permeability could be interpreted as reduced hindrance/restriction parallel to tortuous and beaded axons. Conversely, *D_e_*_⊥_ decreased with increasing permeability, as the radial diffusion of water molecules transitioning from the EAS to the IAS became more restricted. Though relatively minor, *p*_2_ gradually increased with axonal membrane permeability.

Given the huge impact of membrane permeability on the accuracy of *f* estimations, it is crucial to acknowledge that these estimates may not always be reliable if the impermeability assumption is not met. One might argue that the presented simulations lack realism, as myelin should be present to prevent exchange between compartments for all axons. However, histological studies (Coelho et al., 2018) suggest that WM voxels typically contain a mix of myelinated and non-myelinated axons. Although myelinated axons comprise the majority, non-myelinated axons represent a considerable proportion and their prevalence increases in pathological conditions. Additionally, some WM voxels may contain other permeable structures such as dendrites and/or somas, for instance when GM and WM substrates mix. In order to effectively predict microstructural parameters in GM, models such as NEXI (Jelescu et al., 2022; Olesen et al., 2022; Uhl et al., 2024, 2025) use the dMRI signal at multiple diffusion times to estimate the exchange time *t_ex_* between the different compartments, linked to the permeability. However, NEXI is limited to GM and there is no study about the effect of different populations with different permeabilities on this parameter. An attempt has been made with eSANDIX (Olesen et al., 2022) to describe a mixture of permeable and impermeable sticks but the model is very degenerate which compromises the precision of estimated parameters. Describing such substrates remains challenging as the minimal number of parameters required might be too high to avoid degeneracies with the available data.

It is important to note that the effect of increasing permeability on SMI estimates observed in this analysis corresponded to a change in membrane permeability without geometrical or spatial occupancy changes. In other words, the effect does not reflect the scenario of myelin loss, which also translates into an increase in EAS. In studies investigating pathological conditions associated with myelin loss (Fieremans et al., 2016; Guglielmetti et al., 2016; Jelescu, Zurek, et al., 2016; Liao et al., 2024), it was observed that *D_e_*_⊥_ (estimated using SMI, DKI or WMTI) increased, contrasting with our simulations in this part. This suggests that the expansion of the EAS following myelin loss, which leads to an increase in *D_e_*_⊥_, has a more dominant effect than the increased permeability of demyelinated axons, which was found to decrease *D_e_*_⊥_ in our analysis.

### 4.5 Astrocyte membrane permeability

Although the SM does not explicitly account for cellular components other than axons, SMI was expected to be sensitive to the presence of astrocytic processes and to potentially misclassify them as axons. This effect was, indeed, observed, particularly when astrocytes were impermeable. At zero permeability, *f* was overestimated compared to its GT (which considered only axons), as anticipated, with estimated values even slightly exceeding AVF+AsVF, which incorporated both axonal and astrocytic volume fractions. Furthermore, *p*_2_ was underestimated relative to its GT (which again considered only axons), reflecting the greater orientation dispersion of astrocytic processes compared with axons. At low GT *p*_2_ values, our previous investigation has shown that *f* tends to be overestimated, which may further explain why the estimated *f* exceeded the AVF+AsVF in this case. As the membrane permeability of astrocytes increased, astrocytic water gradually became classified as EAS and the estimated axonal *f* and *p*_2_ converged towards their GT values.

Compartment-specific diffusivity estimates were relatively stable over astrocyte membrane permeability values. In the noiseless case, one noticeable trend was a gradual increase in *D_a_* and decrease in *D_e_*_∥_ and *D_e_*_⊥_, which can be explained by the shift of astrocytic water from being associated with the axonal compartment (permeability = 0) to being associated with the extracellular water (permeability $=10^{-4}$ m/s). Indeed, as astrocytic processes are shorter, branched and overall less straight than axons, their association with axonal water would decrease *D_a_*. Conversely, when they contribute to the extra-axonal compartment, they make for a more tortuous medium, hence a decrease in the apparent *D_e_*_∥_ and *D_e_*_⊥_. This influence is attenuated by the addition of noise. The GT *D*_∥_ and *D*_⊥_ were overall very stable (with a slight decrease with permeability), confirming that the changes in compartment diffusivities are driven by shifts in the compartment to which astrocytes are assimilated, not affecting much the weighted average of axial or radial diffusivity in the voxel.

These observations can be compared to findings by Badaut et al. (2011), who reported a marked reduction in ADC following the knockdown of aquaporin-4 water channels, which decreased astrocytic membrane permeability. As astrocyte morphology was assumed to remain unchanged in that study, the reduction in ADC was attributed solely to the decrease in permeability, indicating that higher permeability facilitates overall water mobility. In our simulations, the slight increase in *D*_∥_ and *D*_⊥_ with lower astrocyte membrane permeability was much smaller than what was observed by Badaut et al. (2011). This attenuated response may result from the relatively low astrocytic volume fraction in our substrates, though they were reported to be as such in deep WM (1% soma volume fraction and 6% processes volume fraction (Coelho et al., 2018)). Furthermore, although efforts were made to preserve cell morphology, the knockdown of aquaporin-4 water channels may have disrupted global cellular homeostasis. This disruption could have induced swelling in surrounding neurons, leading to a more substantial decrease in the ADC.

Overall, as astrocytes in physiological conditions are known to be quite permeable, these results confirm typical SM hypotheses, where the EAS is assumed to lump contributions from actual extracellular space and other non-axonal structures (Fieremans et al., 2011). As a result, this suggests that the SM extra-axonal compartment may absorb contributions from glial cells, and EAS diffusivities could therefore be sensitive to glial-related alterations.

It is important to note that this study isolated the effects of astrocyte membrane permeability without altering glial density or fundamental morphology. Structural and density-related alterations could exert a more pronounced effect on EAS diffusivities and impact SMI estimates more markedly than the permeability driven changes reported here. This hypothesis aligns with prior research linking EAS diffusivities to neuroinflammation and glial proliferation in the WM (Falangola et al., 2014; Jelescu & Fieremans, 2023; Liao et al., 2024; Pavan, Alemán-Gómez, Jenni, et al., 2025; Pavan, Wang, et al., 2025). Future research could explore the influence of diverse astrocytic morphologies on SMI estimates. Extending these simulations to include oligodendrocytes and microglia, in addition to varying astrocytic phenotypes, would be insightful for characterising the full spectrum of non-neuronal contributions to the diffusion signal.

### 4.6 Myelin volume fraction

The inclusion of myelin was also expected to challenge the SMI estimations, as this compartment is not explicitly represented in the model. Since myelin is assumed to not contribute to the overall MRI signal due to the short *T*_2_ of myelin water, it is essential to distinguish between *f*, which accounts only for signal-bearing compartments, and the AVF+MVF, which reflects the total intracellular volume, as previously discussed. When the MVF is properly factored out in defining the effective GT, all SMI parameter estimates remained relatively close to their GT values in our results. We observed a substantial underestimation of *p*_2_ and a marginal overestimation of *D_a_*. These biases are likely driven by the low packing density rather than the presence of myelin itself. The estimated *f* lay between the AVF (computed without accounting for myelin) and the AVF+MVF, as expected, although it was slightly overestimated compared to its GT.

Regarding the diffusivity estimates, a decrease in both *D_e_*_∥_ and especially *D_e_*_⊥_ (both the effective GTs and their estimates) could be observed, whereas *D_a_* was rather constant. The large decrease in *D_e_*_⊥_ with higher MVF was expected, as this variable has been considered the main biomarker for myelination and demyelination in countless studies (Aung et al., 2018; Coronado-Leija, Lee, et al., 2024; Fieremans et al., 2013; Guglielmetti et al., 2016; Huber et al., 2021; Jelescu, Zurek, et al., 2016; Jelescu et al., 2015; Liao et al., 2024; Pavan, Alemán-Gómez, Jenni, et al., 2025; Zhou et al., 2020).

### 4.7 Diffusion time

A formal error metric between the SMI estimates and the GT diffusivities was not computed due to a fundamental discrepancy in how diffusion time is defined in the estimated and GT values. Specifically, the effective diffusion time for the diffusion-weighted measurements is given by Δ−δ/3 , whereas for GT diffusivity, particle trajectories accumulate diffusion over the entire echo time, defined as Δ+δ+5 ms.

Taking into account this mismatch, we can observe that *f*, *D_e_*_∥,_ and *D_e_*_⊥_ converged towards their GT at longer diffusion times. On the other hand, the underestimation of *p*_2_ was constant and *D_a_* became increasingly overestimated.

At longer diffusion times, a “coarse-graining” effect (Novikov & Kiselev, 2010) is expected. As water molecules travel over longer times and distances, their motion averages out the complex microscopic irregularities of the axons (such as local bumps or bends). This physical “smoothing” allows the model to ignore the fine structural noise and more reliably estimate the overarching, large-scale geometry of the tissue. This coarse-graining process aligns with effective medium theory (Kiselev, 2017; Novikov & Kiselev, 2010), which dictates that measured diffusivity is expected to decrease gradually with increasing diffusion time, eventually converging to a long-time limit (*D*_∞_). This time-dependence arises from the short-range structural disorder inherent to complex geometries (Kiselev, 2017; Novikov & Kiselev, 2010) as previously demonstrated in numerical substrates (Andersson et al., 2020, 2022; H. H. Lee et al., 2020; Nguyen-Duc et al., 2026). The effect is particularly pronounced for axial diffusivities when the diffusion length probed aligns with the typical spatial scale of axonal irregularities, a phenomenon well-documented in experimental studies (Abdollahzadeh et al., 2025; Fieremans et al., 2016).

The GT diffusivities in our simulations exhibited this expected decrease. However, the variation was subtle for the EAS diffusivities, indicating that they had already closely approached their respective *D*_∞_ values within the evaluated time window. When comparing the diffusivity estimates against this physical baseline, *D_a_* and *D_e_*_∥_ followed this expected downward trend, but *D_e_*_⊥_ surprisingly increased with diffusion time, though it still converged towards its GT at longer times. This implies that the observed increase in estimated *D_e_*_⊥_ is not a reflection of true physical time-dependence, but rather a recovery from a systematic underestimation bias inherent to the model at shorter times.

Regarding the structural parameters, the estimated *f* progressively decreased with diffusion time, converging toward the time-independent GT. Conversely, the GT *p*_2_ increased as a function of diffusion time. As the diffusion length expanded, the metric became more sensitive to the macroscopic alignment of the axons rather than their microscopic beading and undulation. The estimated *p*_2_ increased at a comparable rate but consistently fell short of the GT, maintaining a constant estimation gap across all time points.

Overall, longer diffusion times generally yielded more accurate SMI estimates, with the notable exception of *D_a_* and *p*_2_. Counter-intuitively, *D_a_* achieved its highest estimation accuracy at the shortest evaluated diffusion time. This discrepancy arises because the GT *D_a_* successfully disentangles microscopic axonal irregularities from macroscopic dispersion, resulting in a pronounced time dependence driven by beading and undulation. In contrast, the SMI framework assumes a substrate of dispersed, straight cylinders and does not explicitly model undulation. As a result, the SMI artificially absorbs the effects of undulation into its macroscopic dispersion parameter, forcing its estimated *D_a_* to remain roughly constant across varying diffusion times.

Beyond these microstructural considerations, pushing to longer diffusion times also introduces practical experimental trade-offs. It is important to note that *T*_2_ relaxation was not accounted for in our simulations or in SMI (though an extension of SMI could do so (Coelho et al., 2022; Lampinen et al., 2020; Veraart et al., 2018)). In a PGSE design, the echo time is coupled to the diffusion time, and thus the SNR decreases with longer diffusion times, which can in turn reduce estimation accuracy. Therefore, an optimal balance must be achieved, where water molecules probe the substrate for a sufficiently long time while maintaining an adequate SNR. Previous work optimised SMI protocols with diffusion times in the order of 30 to 50 ms (Coelho et al., 2022).

### 4.8 Noise

Across all figures comparing results with and without noise, we observed that decreasing SNR led not only to an increased standard deviation (reflecting lower precision) for all parameter estimates, but also to consistent biases, most notably in *p*_2_ and *D_e_*_∥_. At lower SNRs, *p*_2_ was systematically underestimated, while *D_e_*_∥_ was consistently overestimated. In contrast, the parameter *f* remained largely unbiased across all SNR levels. As reported by Reisert et al. (2017), this behavior is expected: volume fractions can be estimated reliably from high-quality data, whereas diffusivity estimates tend to be noisier and more biased across acquisition schemes. Furthermore, Coelho et al. (2022) identified *D_e_*_∥_ as the most noise-sensitive parameter, being the most difficult to estimate and therefore the most influenced by training data. Despite these biases, the same study concluded that SMI remains sufficiently robust to capture biological variability across WM regions (and potentially pathological variability) making it “good enough” for practical use. Indeed, such biases would not necessarily preclude sensitivity to specific biological phenomena (e.g., intra-axonal diffusivity for beading) and thus group-comparison studies, as relative differences between groups may still be biologically meaningful. Regarding *p*_2_, our findings are consistent with prior work showing that low SNR values (below 15) substantially hinder the accurate estimation of the fanning extent (Sotiropoulos et al., 2012). Although our simulations only considered SNR levels of 50 and 100, a clear bias in *p*_2_ was already evident, suggesting that this effect would likely be even more pronounced at lower SNRs (typically close to 30 in WM). Our analysis thus extends previous work by explicitly revealing the direction of the bias: *p*_2_ tends to be underestimated, while *D_e_*_∥_ is overestimated in the presence of noise.

While the primary analyses in this study utilized Gaussian noise, analogous results generated using Rician noise are provided in the Supplementary Materials (Supplementary Figs. S5–S12). The parameter trends observed under Gaussian noise were fully consistent with those obtained using Rician noise. This indicates that the noise floor induced by the Rician distribution did not significantly affect the SMI estimates under the tested conditions.

### 4.9 Summary

Overall, the amplitude of change in SMI estimates when varying parameters (summarized in Figs. 12 and 13) indicates that:

1. The estimated intra-axonal water fraction *f* increased as expected with stronger beading, higher axonal packing density, and greater myelin volume. It was overestimated with stronger beading and high axonal dispersion, underestimated with high axonal membrane permeability.
2. The effective orientation dispersion index *p*_2_ was underestimated across most scenarios. This bias within the SMI framework was the most pronounced at low axonal packing densities, but was also exacerbated by stronger beading and the presence of impermeable astrocytes. Surprisingly, *p*_2_ was on the contrary overestimated in the presence of two crossing fibre populations.
3. The effective intra-axonal diffusivity *D_a_* was closely tied to axonal morphology, decreasing with stronger beading and undulation. Furthermore, it became overestimated by SMI in most conditions, but particularly when multiple crossing populations were present.
4. The effective parallel extra-axonal diffusivity *D_e_*_∥_ was strongly affected by axonal membrane permeability, but also by packing density, dispersion, and undulation strength. Its estimate was also the most noise-sensitive parameter, showing systematic overestimation at low SNR levels (SNR = 50). At higher SNR levels, it was consistently accurately estimated.
5. The effective perpendicular extra-axonal diffusivity *D_e_*_⊥_ was mainly sensitive to axonal packing density, axonal membrane permeability, and myelin volume fraction, making it a potential biomarker for (de)myelination, but also to undulation. Its SMI estimate was also consistently accurate with respect to the effective GT.

**Fig. 12.**
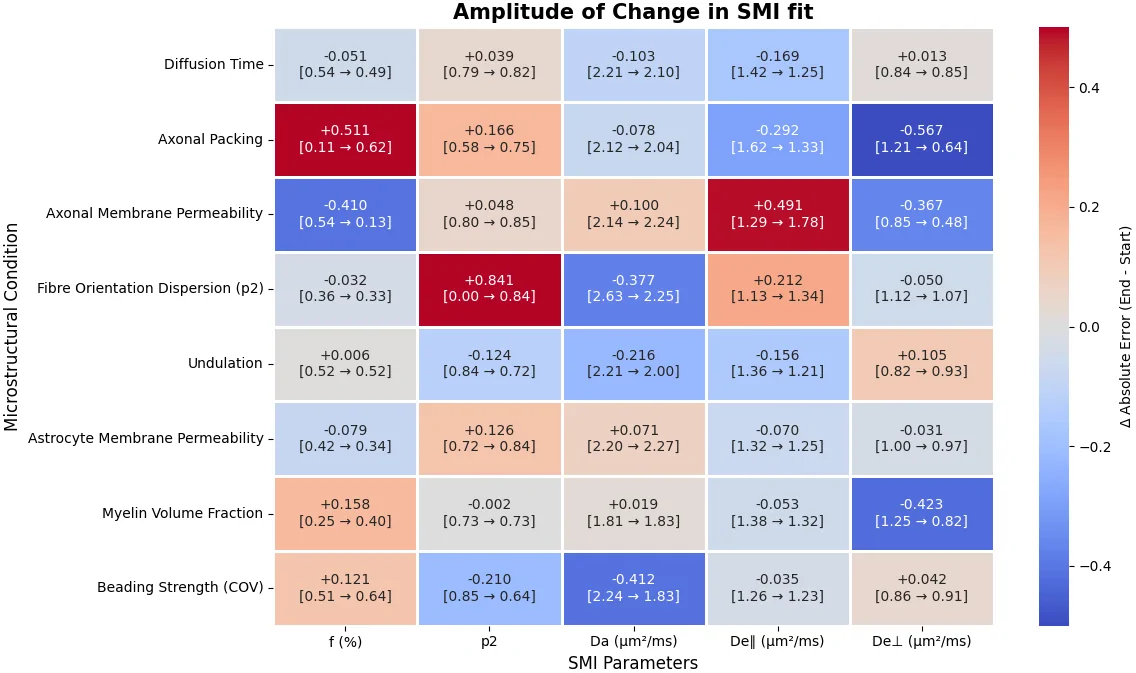
Amplitude of change in SMI fit across the range of the varied parameter. It was computed for the first and last values of the varied parameter (shown in parentheses). These results correspond to noiseless data.

**Fig. 13.**
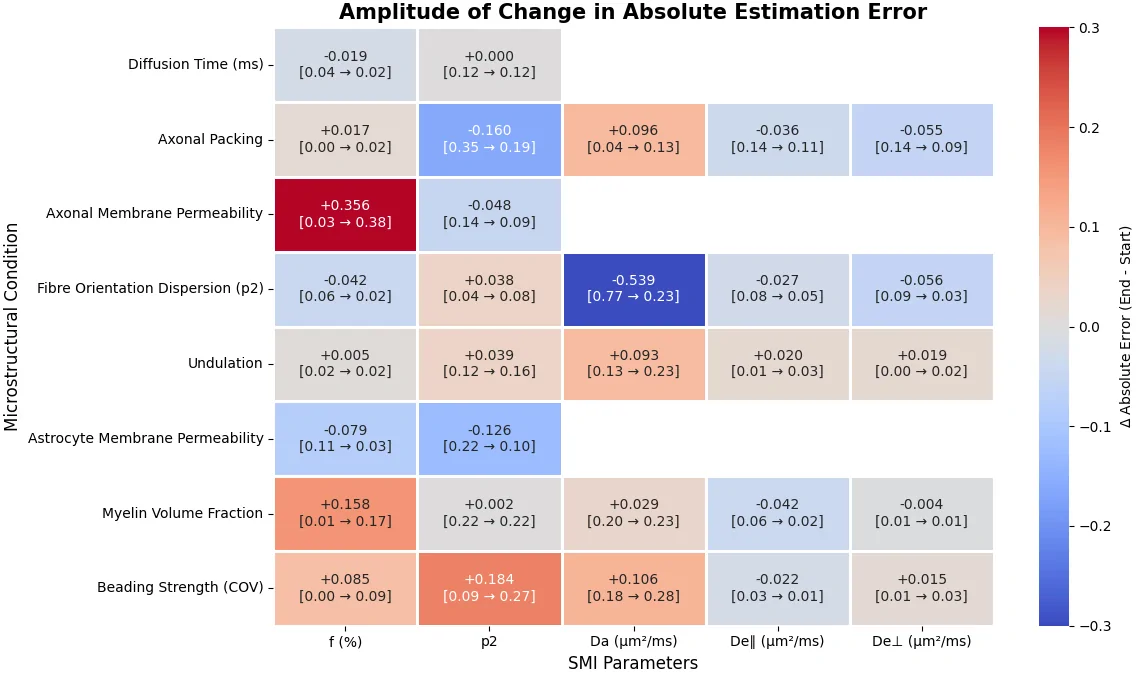
Change in the error of each SMI estimate across the range of the varied parameter. The error was defined as $\left|\tilde{s}-s\right|$, where $\tilde{s}$ denotes the SMI estimate and *s* the corresponding GT. The error was computed for the first and last values of the varied parameter (shown in parentheses), and their difference is reported. This difference is negative when the variation decreases the SMI error (i.e., improves accuracy) and positive when it increases the error. A value close to zero indicates that the parameter had little influence on the SMI estimation error. No values could be obtained when membranes were permeable, as there was no GT for all three diffusivity parameters. When evaluating the impact of diffusion time, the errors could not be assessed for diffusivities because the GT diffusivities and SMI estimates were obtained at mismatched diffusion times. These results correspond to noiseless data.

While these biases compromise the quantitative accuracy of individual parameter estimates, they do not necessarily preclude high sensitivity to specific biological microstructural features and relative differences between groups. Provided the bias does not affect the trend between the estimated and GT values, the estimates could still track real biological variation, even if their absolute values remain inaccurate. This distinction matters for group-comparison studies: absolute SMI values should be interpreted with caution, but relative differences between groups may still be biologically meaningful.

## 5 Limitations

MC simulations were conducted within voxels measuring 150 μm in width, with walkers initialised inside a central cube of 110 μm. While this configuration reduces the likelihood of walkers reaching the voxel boundaries, it does not fully eliminate it. Encounters with the mirror boundaries can induce artificial turning behavior, potentially affecting the *p*_2_ estimation.

Additionally, though the impact of each variable on the SMI estimates was assessed, we did not investigate how they impact them jointly. For example, cell membrane permeability may affect the results even more when the packing density is higher. Future work could attempt to assess the combined effects of these parameters to better capture their interdependencies.

Another limitation of this work is that variables could not always be varied fully independently. In particular, increasing packing density also altered axonal morphology by increasing beading amplitude, likely due to axonal shrinkage to accommodate the narrower inter-axonal gaps that become more frequent at higher packing densities. This coupling made it difficult to isolate effects that were directly attributable to packing density alone, as opposed to the accompanying changes in beading.

Furthermore, the simulation of myelin did not include diffusing water particles between successive myelin layers nor compartment-specific *T*_2_ relaxation (Harkins & Does, 2016; Lin et al., 2018). As a result, increasing myelin volume effectively reduced extracellular space and increased relative *f*, without capturing myelin’s restricted diffusion and potential exchange between myelin and extra-axonal water. Thus, the simulation does not fully model myelin properties, and future work incorporating these mechanisms would be needed to isolate myelin’s specific contribution to SMI estimates.

As noted in the diffusion time analysis, a fundamental discrepancy exists in how diffusion time is defined for the diffusivity estimates versus the GT values. Given Δ=55.5 ms, the effective diffusion time for SMI is defined as Δ−δ/3 (yielding 50 ms), whereas the GT diffusion time is defined as Δ+δ+5 ms (yielding 77 ms). It is important to emphasize that this temporal discrepancy was inherent to our overall experimental design, except in the analysis that was specific to time-dependence. Thus, the overestimation of *D_a_* relative to the GT may have been magnified, given that the GT *D_a_* decreases substantially at longer diffusion times. In contrast, the time-dependent decreases in GT *D_e_*_∥_ and *D_e_*_⊥_ were much less pronounced, suggesting their interpretation remains largely unaffected.

## 6 Conclusions and Future Works

Using numerical WM substrates, we were able to assess the variation of the SMI parameter estimations across various substrates, and to identify the conditions to which the model parameters were the most sensitive, but also the ones under which the parameter estimates exhibited the greatest biases. Awareness of potential biases under different conditions is crucial for a reliable interpretation of results in applications to real tissue. The findings revealed that among others, axonal membrane permeability, high orientation dispersion, and low axonal packing density considerably affected the accuracy of the SMI estimations. All of these conditions considerably affected the estimation of *f*, *p*_2_, and *D_a_*. SMI-derived EAS compartment diffusivities, on the other hand, displayed an excellent accuracy in the conditions of our simulations. However, *D_e_*_∥_ was particularly biased with the addition of noise.

In future work, we plan to further enhance the realism of our numerical substrates by incorporating blood vessels in CATERPillar. An updated version of the MC/DC tool with integrated *T*_2_ relaxation could then be used to assess how vasculature-induced inhomogeneities influence diffusion-weighted signals.

## Supplementary Material

Supplementary Material

## Data Availability

The source codes for both the CATERPillar tool and the modified MC/DC tool have been made publicly available on GitHub: https://github.com/jazz031195/CATERPillar and https://github.com/jazz031195/Permeable_MCDS
