# Supplementary material for: Validating the standard model of diffusion MRI in white matter with numerical substrates
Source: Imaging Neurosci (Camb). 2026 Oct 15;4:IMAG.a.1385. doi: 10.1162/IMAG.a.1385 (PMC13673310; doi:10.1162/IMAG.a.1385)
Supplement: Supplementary file 1 [file IMAG.a.1385_supp.pdf]

Supplementary Material

April 28, 2026

Assessing the impact of the number of random walkers in MC/DC

To verify that a density of one random walker per μm^3 (corresponding to a total of 1,330,000 walkers) yields stable and accurate MRI signal estimates, we evaluated the coefficient of variation (COV $= \frac{\mathrm{std}}{\mathrm{mean}}$) of the signal across several walker densities. Because a lower COV indicates greater signal stability, our goal was to determine the density at which the COV plateaus, thereby justifying our choice. To this end, we performed the MC simulations on a substrate containing axons with a packing density of 0.5.

We performed 40 independent MC simulations, each with 133,000 walkers within the IAS and EAS compartments separately. The signal from these independent runs were normalised and then combined in random groups to emulate higher walker densities.

We compared the signal obtained when combining 1, 2, 5, 7, 10, 15, 20, and 30 runs with 133,000 walkers each. For each group, the signal was generated 10 times with random sampling (with replacement), and the mean and COV across these 10 groups.

This procedure was repeated for all walker densities, and the resulting mean and COV values were averaged over 100 repetitions. The final results (Supplementary Figure S1) present the mean powder-averaged DWI signal and corresponding COV as functions of walker density and b-value. At a density of 1 walker/μm^3, the COV of the normalised powder-averaged signal was below 0.002 for the IAS and 0.02 for the EAS. The highest COV in the EAS occurred at a b-value of 5 ms/μm^2, consistent with expectations since such high b-values are known to attenuate the extracellular signal. The chosen density is marked by an arrow in Figure S1. The elbow point (identified using the KneeLocator library (Satopaa et al., 2011)) is located at 0.70 walkers/μm^3, just before the arrow. In optimisation problems, the elbow typically marks the point beyond which further increases are not worth the additional cost. Selecting the

next density step therefore provides a small safety margin. Thus, our results indicate that choosing one walker per μm^3 was appropriate, as the elbow occurs immediately before this value.

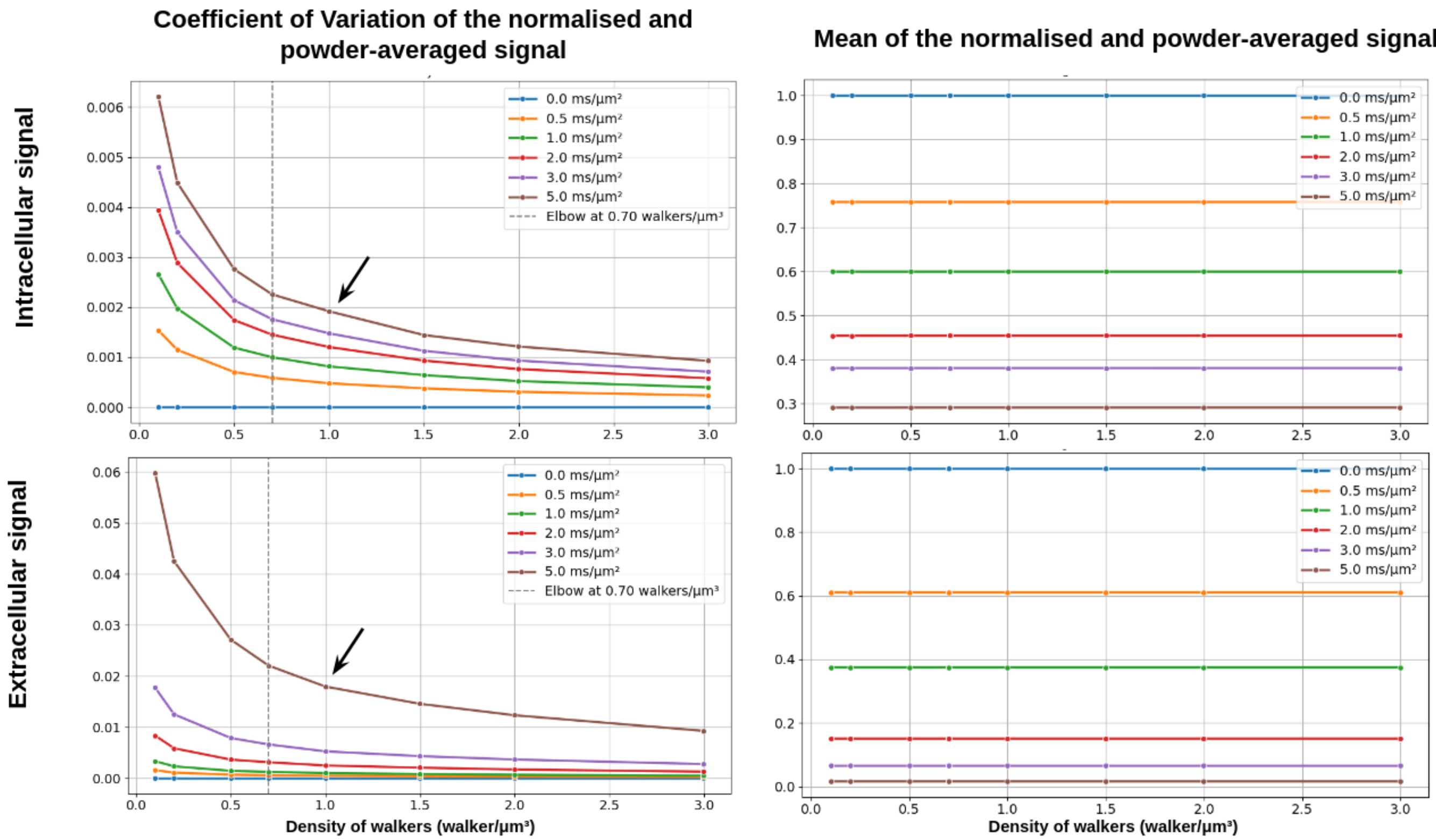

Figure S1: Average coefficient of variation (COV) and mean normalized powder-averaged signal. Mean values and COVs were computed in groups of 10 and recalculated 100 times through resampling. Results are shown for multiple b-values (indicated in the legend) in ms/μm^2. The arrow marks the walker density selected for the MC simulations in this study.

Run-time for substrates of varying orientation distribution

Supplementary Figure S2 illustrates the computational run-times for the substrates employed in the orientation dispersion analysis. These voxels featured a packing fraction of approximately $f = 0.3$ and a voxel length of 150 μm. Notably, the duration of the MC simulations consistently exceeded the time required for substrate generation. Further reductions in run-time are attainable by scaling the hardware allocation beyond 48 cores for the MC simulations and 10 cores for substrate generation.

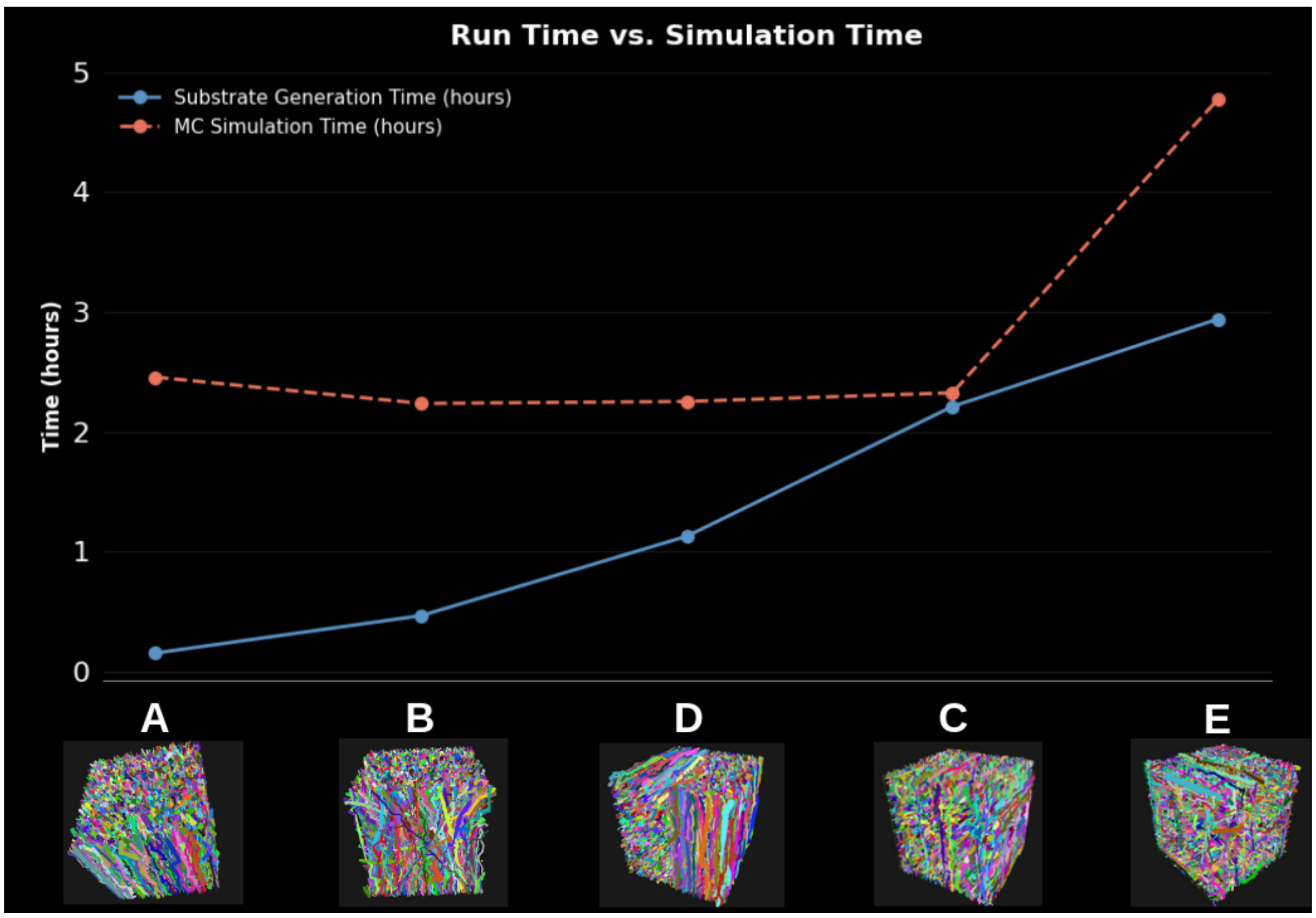

Figure S2: Computational run-times for substrate generation using CATERPillar (10 cores) and MC simulations (48 cores). Each thread used one core and approximately 300 MB of RAM. These substrates were used for the orientation dispersion analysis.

Assessing SMI estimates from substrates generated with same morphological parameters

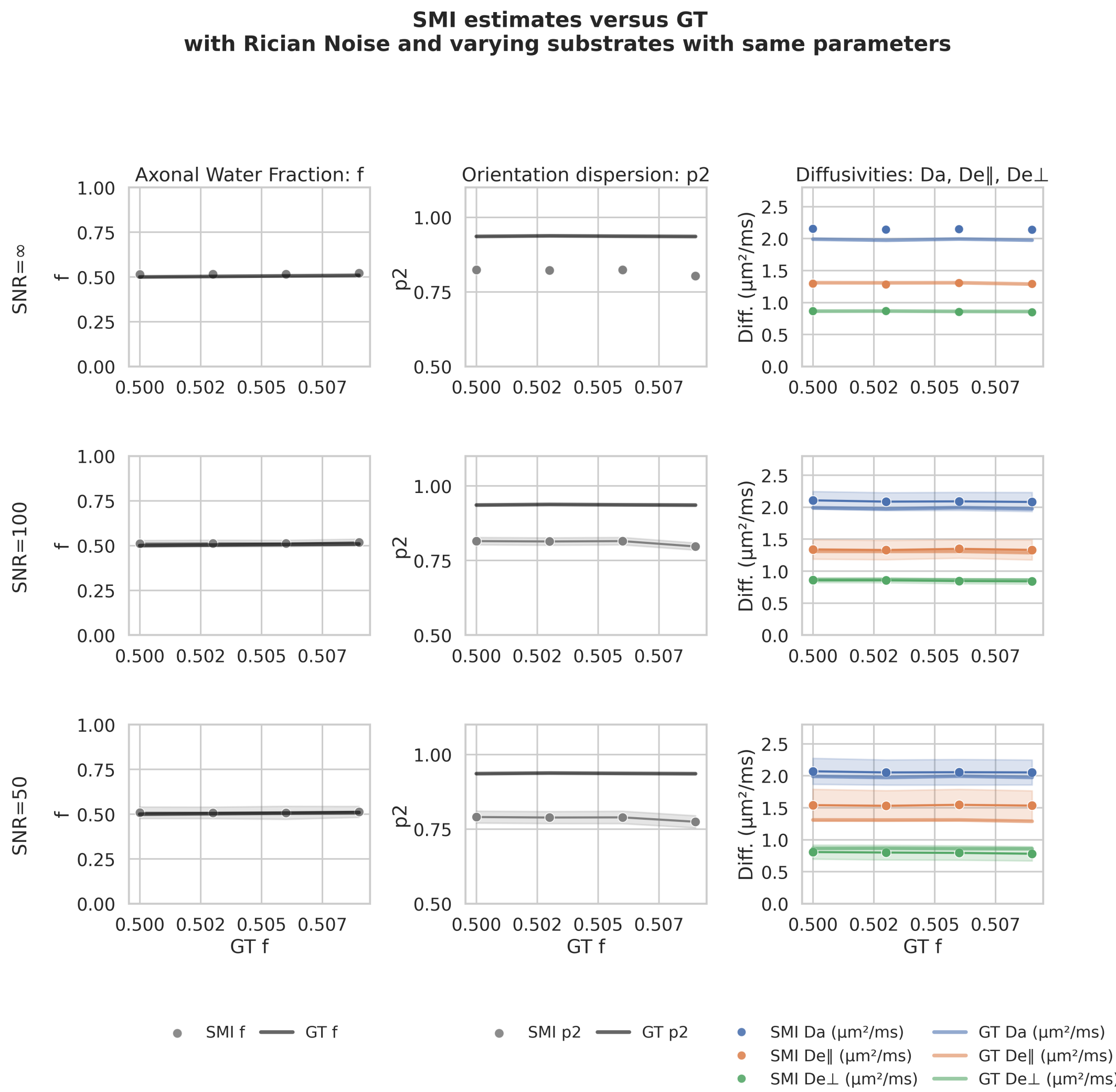

Figure S3: SMI estimates of f, p_2, $D_{\|}$, $D_{e\perp}$, and D_a are shown together with their corresponding GT values for each substrate generated. The parameters for the generation of the substrates are identical. No noise has been added to this data.

Distribution of radii in substrates of varying morphological features

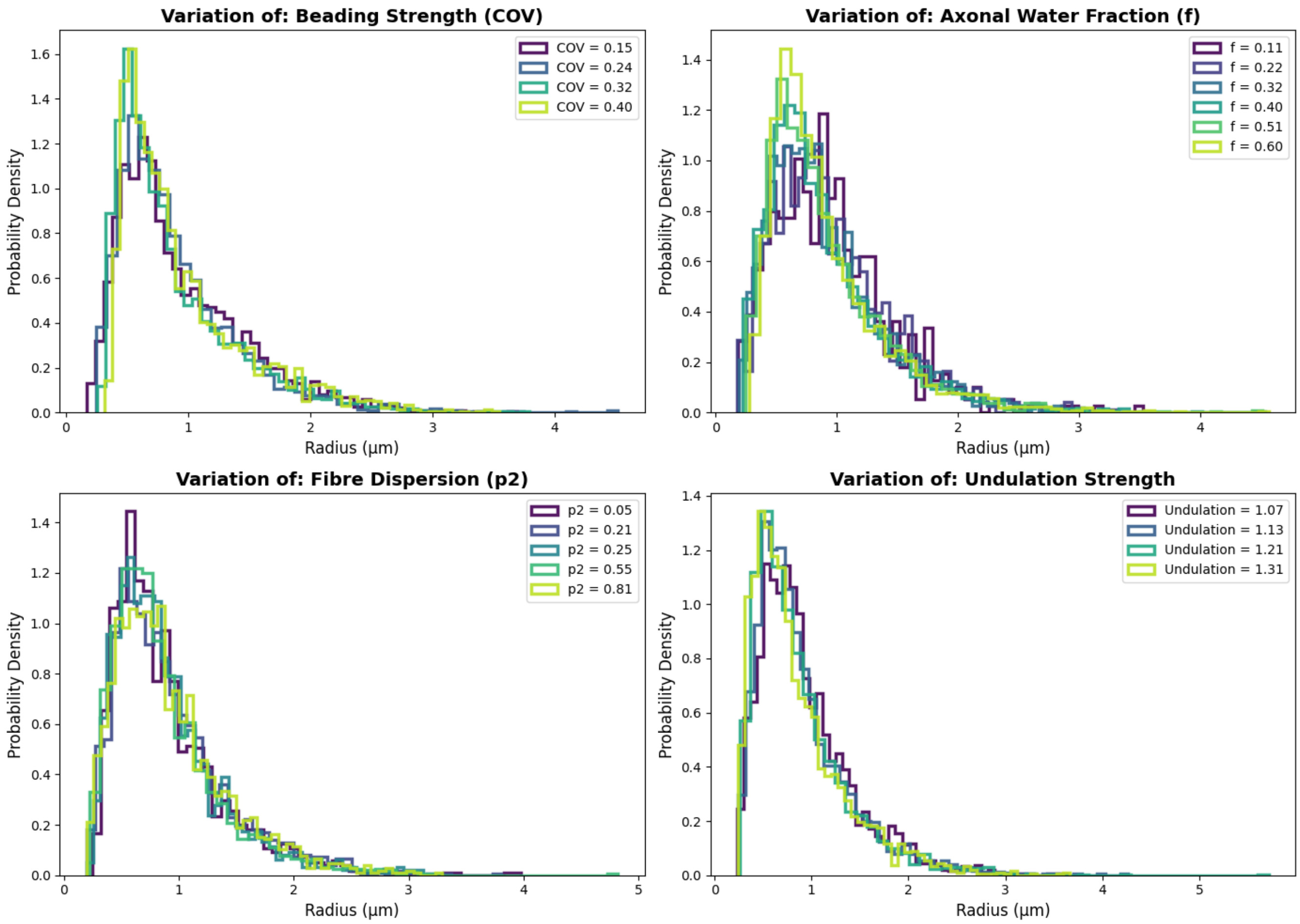

Figure S4: Axonal radius distributions across substrates, generated by independently varying single microstructural features: beading strength (COV), intra-axonal water fraction (f), dispersion (p_2) and undulation. The high degree of consistency across these distributions confirms that subsequent changes in SMI estimates are driven primarily by the targeted microstructural variations, rather than being confounded by differences in axon calibre.

SMI estimates versus GT with Rician noise

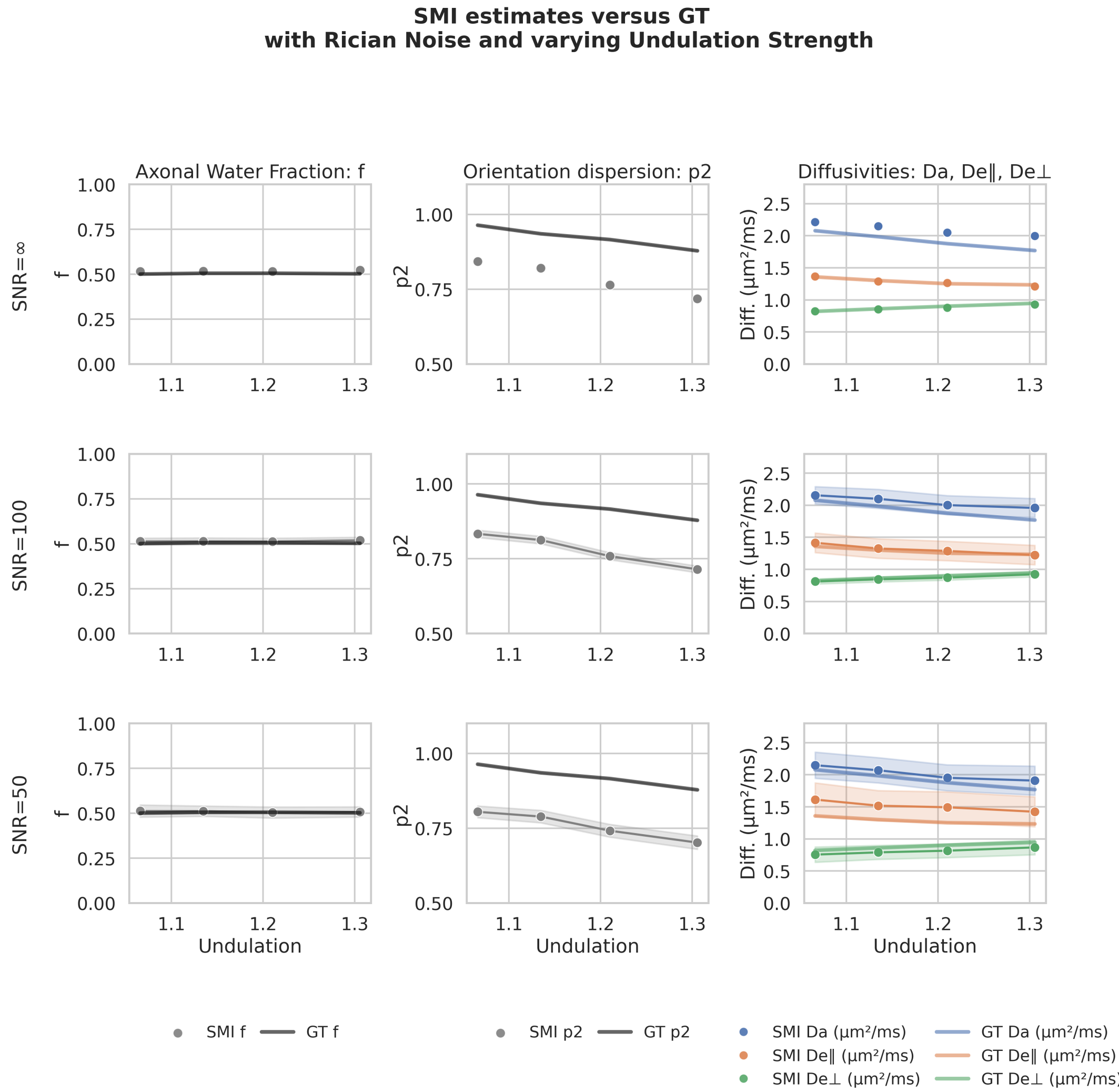

Figure S5: SMI estimates of f, p_2, $D_{e\|}$, $D_{e\perp}$, and D_a are shown together with their corresponding GT values as a function of the mean undulation coefficient for each voxel. When noise is included, the shaded areas represent the standard deviation across 1000 repetitions.

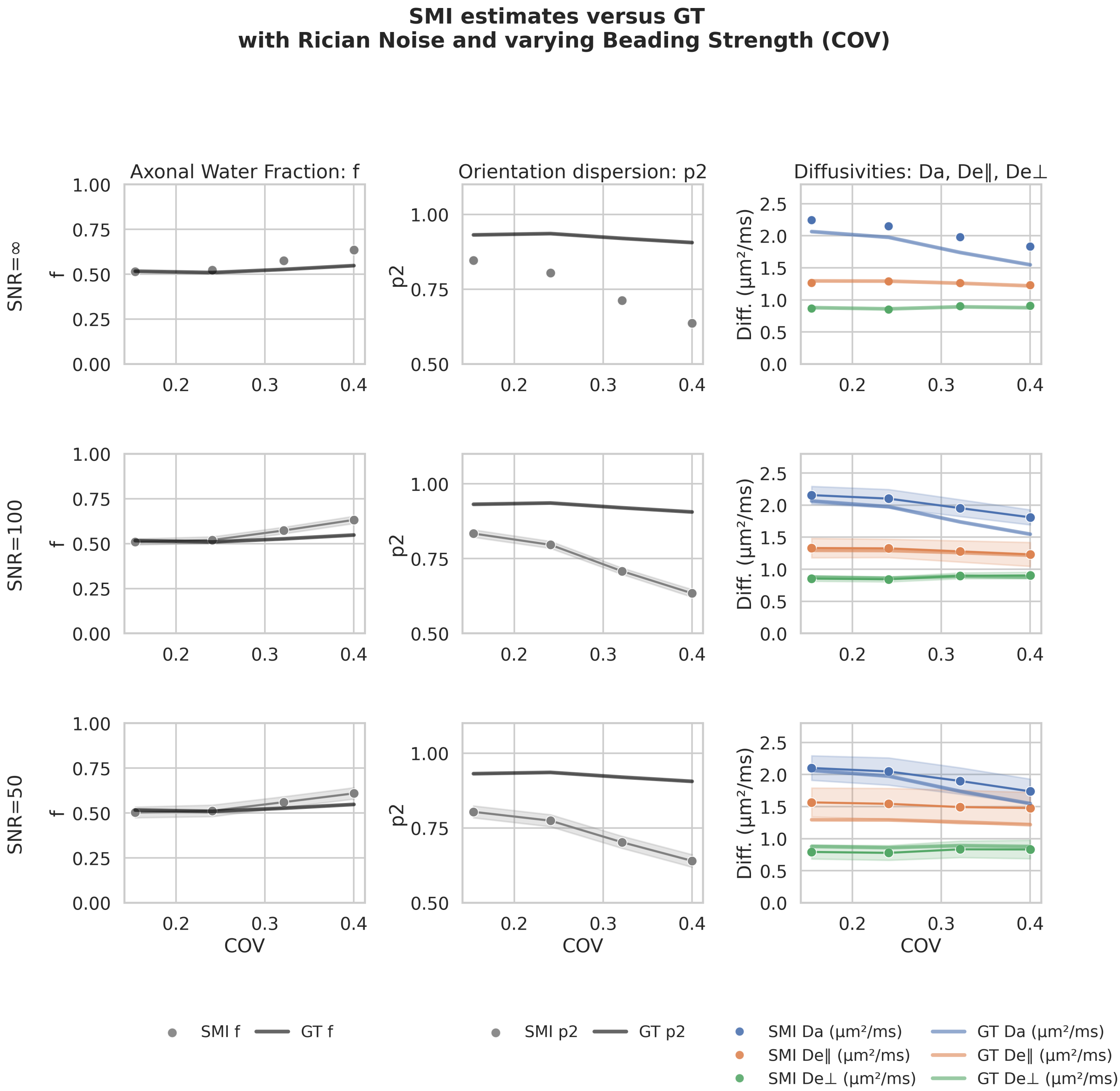

Figure S6: SMI estimates of f, p_2, $D_{e\|}$, $D_{e\perp}$, and D_a are shown together with their corresponding GT values as a function of the mean COV for each voxel. This COV quantified the beading strength of the axons, which was modulated across subjects by varying the parameter σ from 0 to 0.3. The mean COV of the axonal radius was obtained by averaging the COV for all axons within the same voxel. When noise is included, the shaded areas represent the standard deviation across 1000 repetitions.

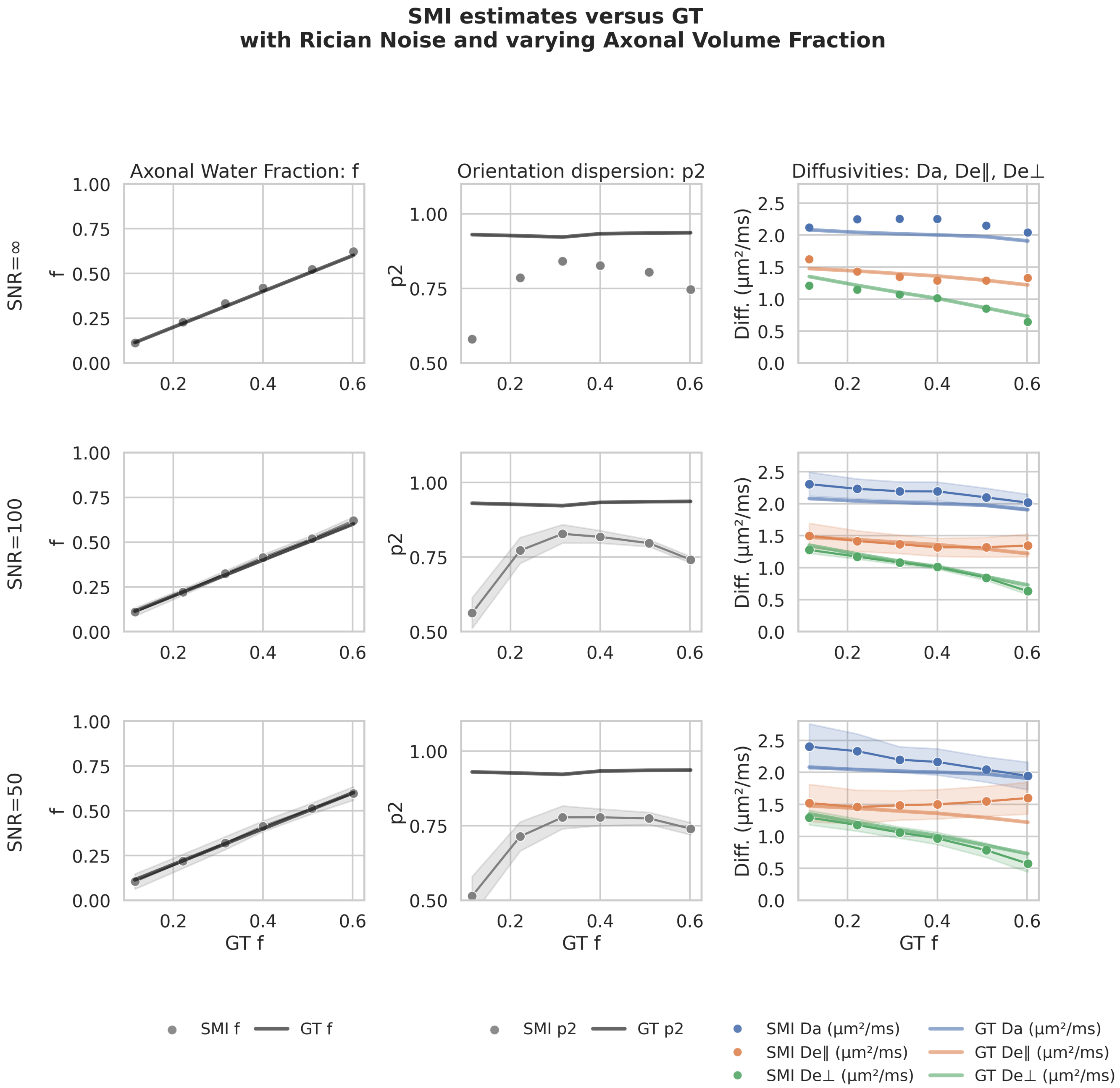

Figure S7: SMI estimates of f, p_2, $D_{e\|}$, $D_{e\perp}$, and D_a are shown together with their corresponding GT values as a function of the GT axonal water fraction f. When noise is included, the shaded areas represent the standard deviation across 1000 repetitions.

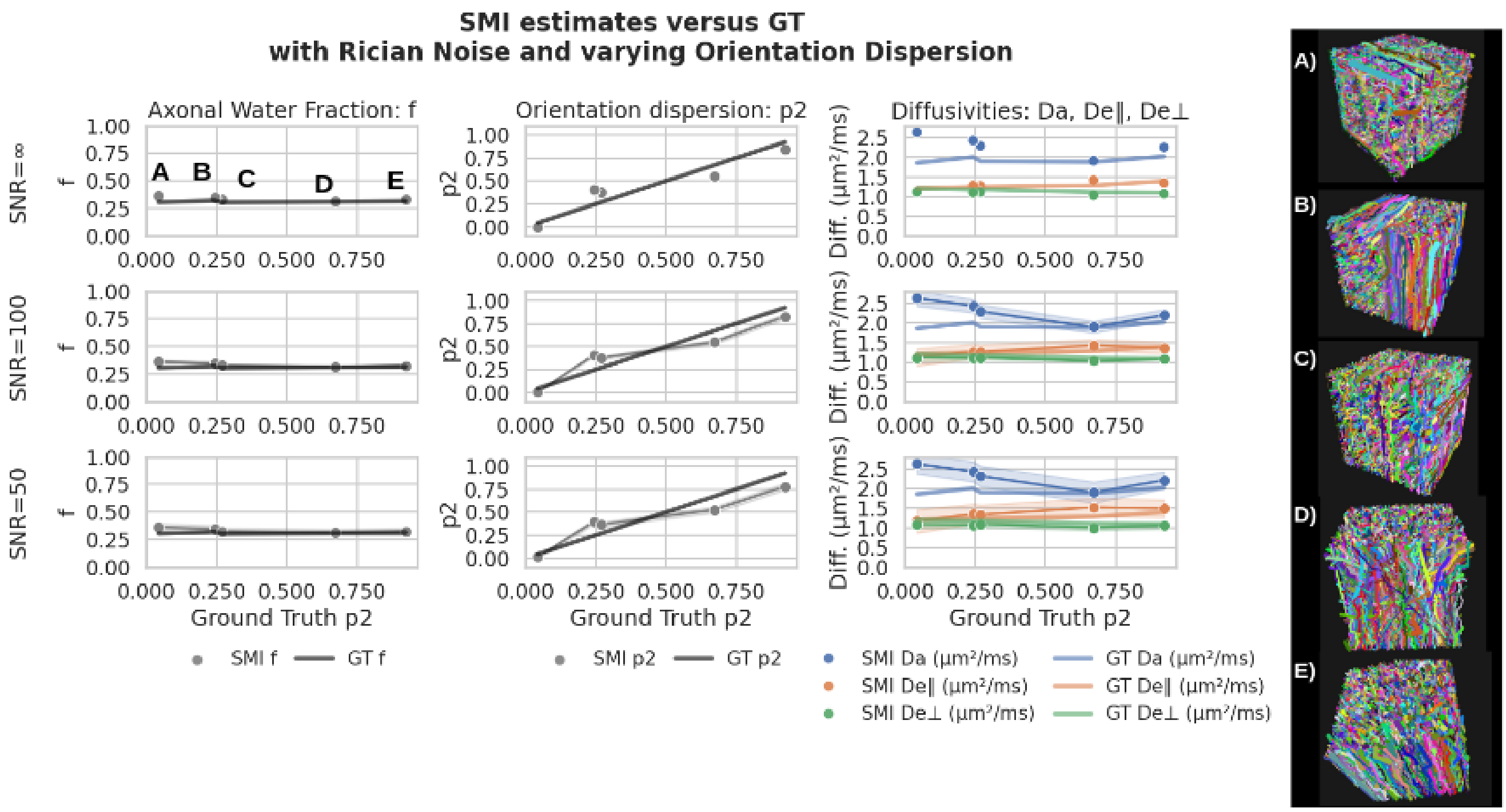

Figure S8: SMI estimates of f, p_2, $D_{e\|}$, $D_{e\perp}$, and D_a are shown together with their corresponding GT values as a function of the mean GT p_2 for each voxel. Substrate (A) contained three perpendicular fibre populations, while substrates (B) and (C) each included two perpendicular populations arranged in sheet-like and interwoven configurations, respectively. Substrate (D) consisted of a single fibre population with fanning, modelled using a Watson distribution, and substrate (E) featured a single population with no fanning. By comparing the SMI parameters from the varying-fODF substrates against the substrate E, we can disentangle the influence of macroscopic orientation dispersion from microscopic morphological changes. When noise is included, the shaded areas represent the standard deviation across 1000 repetitions.

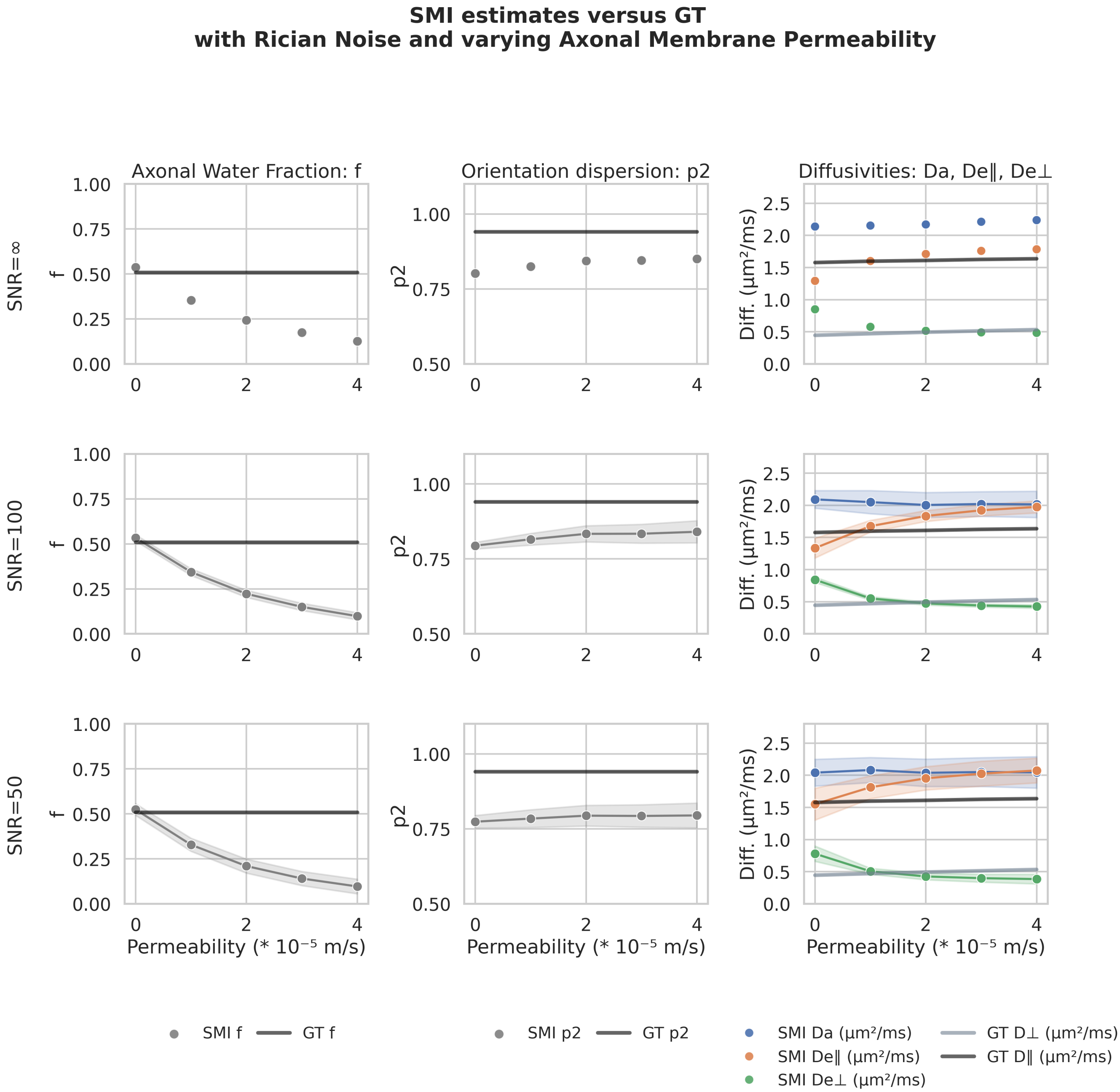

Figure S9: SMI estimates of f, p_2, $D_{e\|}$, $D_{e\perp}$, and D_a are shown together with their corresponding GT values as a function of the membrane permeability of the axons for each voxel. When noise is included, the shaded areas represent the standard deviation across 1000 repetitions.

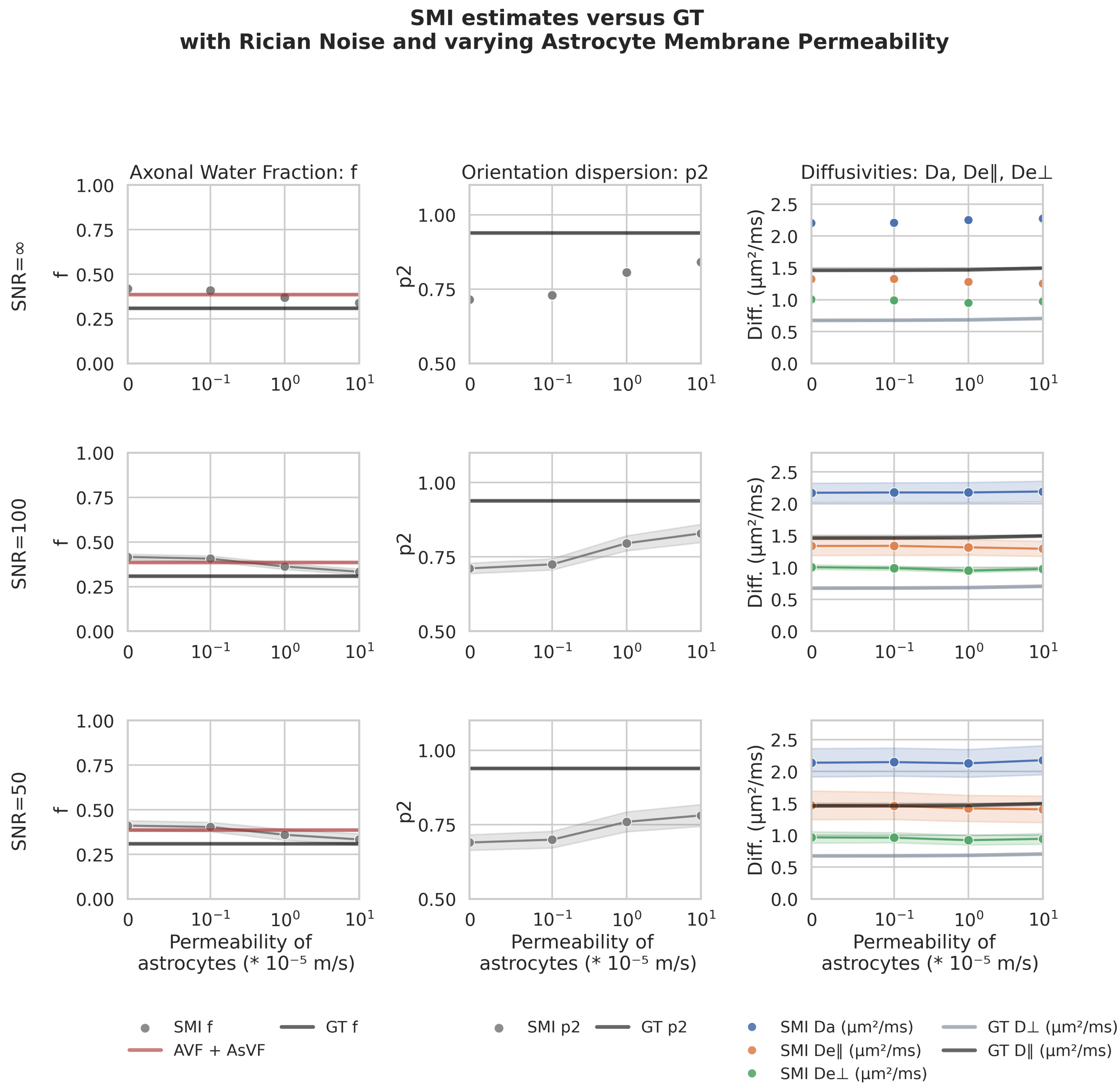

Figure S10: SMI estimates of f, p_2, $D_{e\|}$, $D_{e\perp}$, and D_a are shown together with their corresponding GT values as a function of the membrane permeability of the astrocytes for each voxel. The astrocytes' soma and processes took 1% and 6% of the total voxel volume. Additionally to GT f, the SMI f can be compared to AVF + AsVF, which accounts for the volume occupied by both axons and astrocytes. When noise is included, the shaded areas represent the standard deviation across 1000 repetitions.

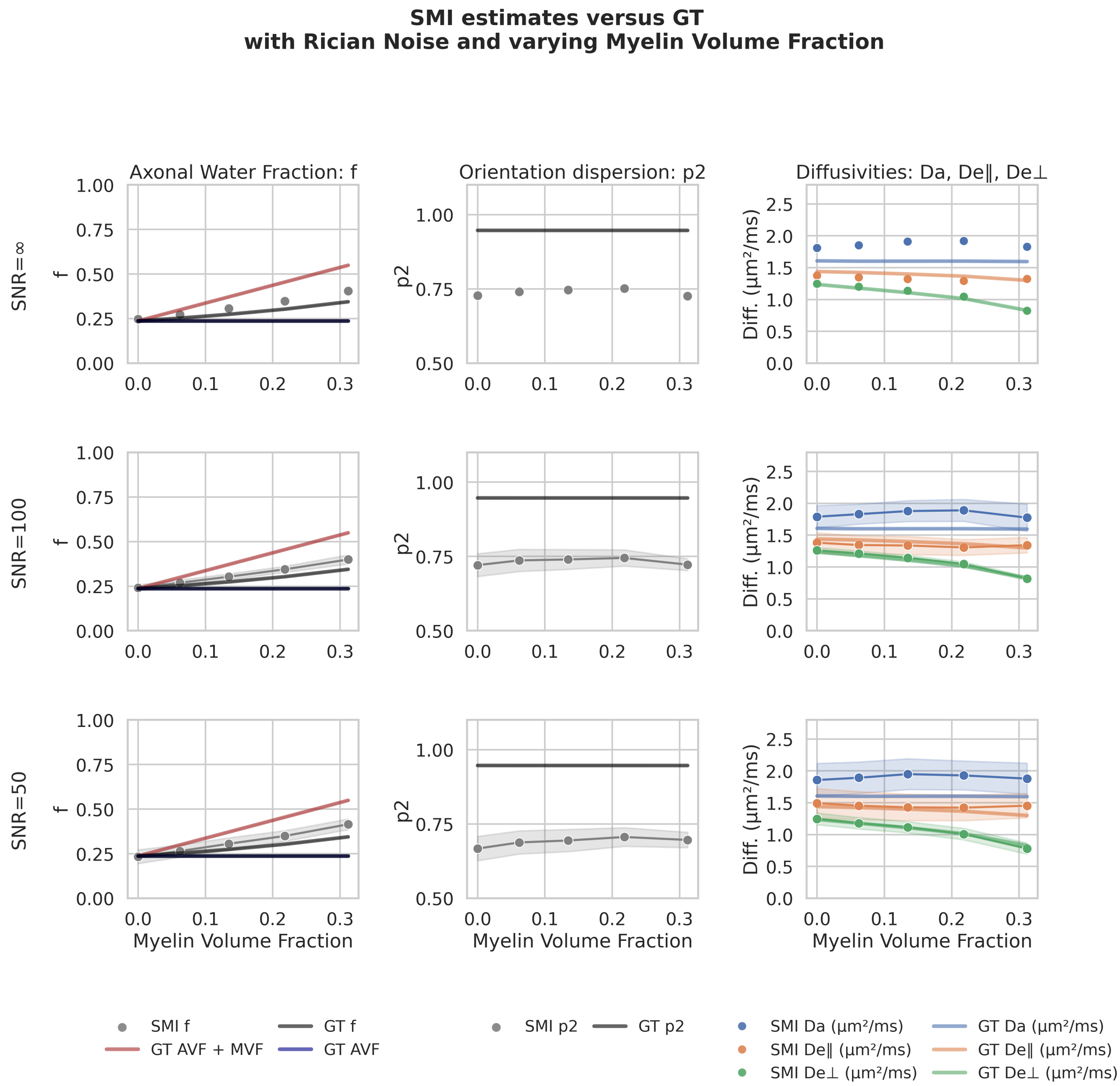

Figure S11: SMI estimates of f, p_2, $D_{e\|}$, $D_{e\perp}$, and D_a are shown together with their corresponding GT values as a function of the MVF for each voxel. GT f takes into account the reduced water volume with increasing MVF, thus increases despite a constant AVF. The AVF + MVF represents the real volume fraction of axons with myelin included. The estimated p_2 is particularly low due to the low GT AVF. When noise is included, the shaded areas represent the standard deviation across 1000 repetitions.

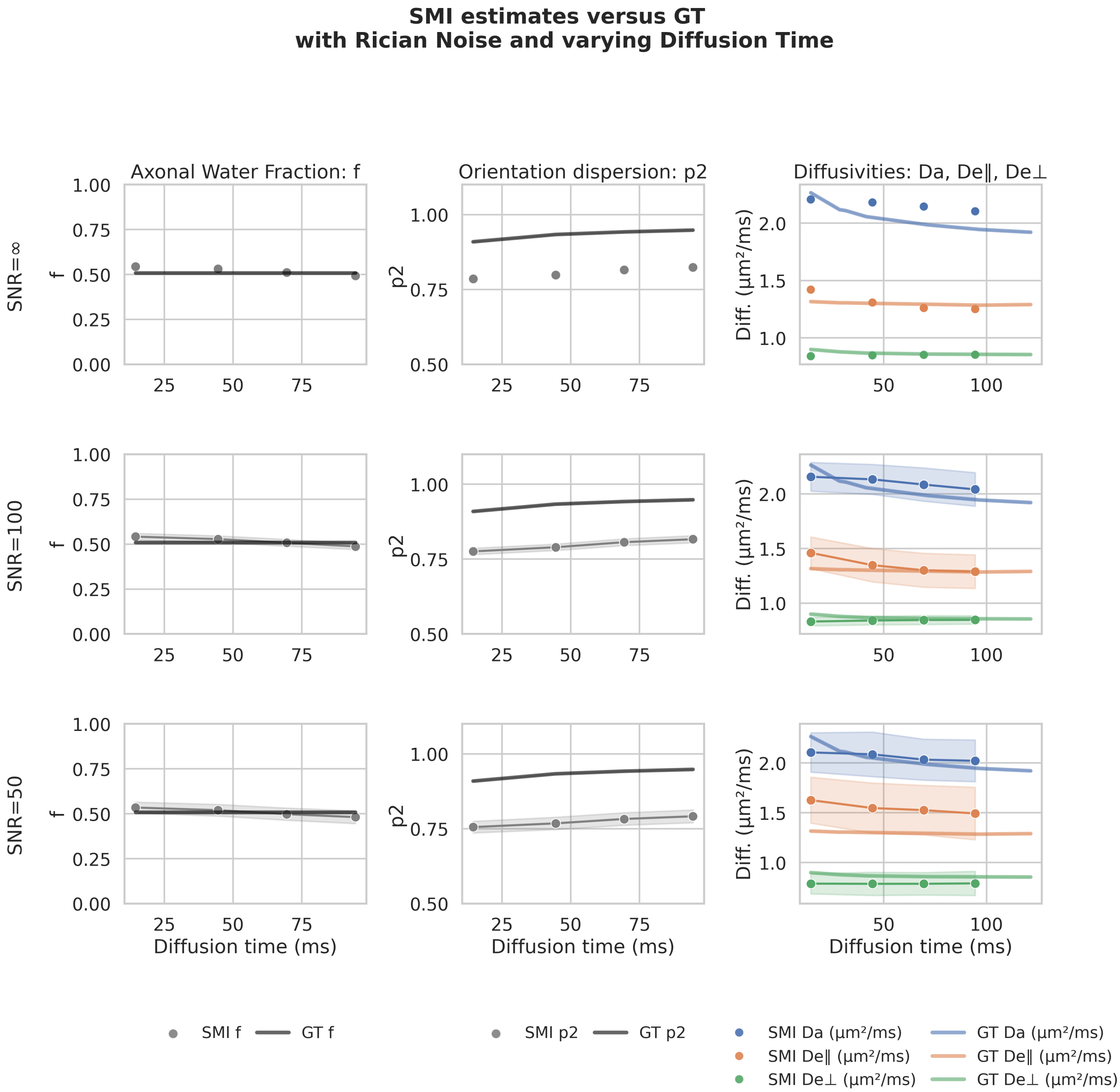

Figure S12: SMI estimates of f, p_2, $D_\parallel$, $D_{e\perp}$, and D_a are shown together with their corresponding GT values as a function of the diffusion time for each voxel. The diffusion time for SMI estimates and GT p_2 was defined as $\Delta - \frac{\delta}{3}$ whereas for the GT diffusivities, it was defined by TE though $TE > \Delta - \frac{\delta}{3}$. When noise is included, the shaded areas represent the standard deviation across 1000 repetitions.

References

Satopaa, V., Albrecht, J., Irwin, D., & Raghavan, B. (2011). Finding a kneedle in a haystack: Detecting knee points in system behavior, 166–171. https://doi.org/10.1109/ICDCSW.2011.20
